# Lipids as Targets for Renal Cell Carcinoma Therapy

**DOI:** 10.3390/ijms24043272

**Published:** 2023-02-07

**Authors:** Bisera Stepanovska Tanturovska, Roxana Manaila, Doriano Fabbro, Andrea Huwiler

**Affiliations:** 1 Institute of Pharmacology, University of Bern, Inselspital, INO-F, CH-3010 Bern, Switzerland; 2 Cellestia Biotech AG, Technology Park Basel, Hochbergerstrasse 60C, CH-4057 Basel, Switzerland

**Keywords:** lipids, sphingolipids, glycosphingolipids, eicosanoids, free fatty acids, cannabinoids, cholesterol, kidney cancer, renal cell carcinoma

## Abstract

Kidney cancer is among the top ten most common cancers to date. Within the kidney, renal cell carcinoma (RCC) is the most common solid lesion occurring. While various risk factors are suspected, including unhealthy lifestyle, age, and ethnicity, genetic mutations seem to be a key risk factor. In particular, mutations in the von Hippel–Lindau gene (*Vhl*) have attracted a lot of interest since this gene regulates the hypoxia inducible transcription factors HIF-1α and HIF-2α, which in turn drive the transcription of many genes that are important for renal cancer growth and progression, including genes involved in lipid metabolism and signaling. Recent data suggest that HIF-1/2 are themselves regulated by bioactive lipids which make the connection between lipids and renal cancer obvious. This review will summarize the effects and contributions of the different classes of bioactive lipids, including sphingolipids, glycosphingolipids, eicosanoids, free fatty acids, cannabinoids, and cholesterol to renal carcinoma progression. Novel pharmacological strategies interfering with lipid signaling to treat renal cancer will be highlighted.

## 1. Introduction

Kidney cancer is among the top ten most common cancers to date and accounts for about 3% of adult malignancies [1,2]. Malignant tumors can arise either from the renal parenchyma or the renal pelvis. In children, the most common kidney cancer is nephroblastoma (Wilms tumor), accounting for 1.1% of all kidney cancers [3], while in adults, renal cell carcinoma (RCC) is the most common neoplasm within the kidney. RCC originates from the renal epithelium, specifically from the proximal convoluted tubule and accounts for >90% of cancers in the kidney. The disease encompasses more than 10 histological and molecular subtypes, of which clear cell RCC (ccRCC) is the most common and accounts for most kidney cancer-related deaths [4,5]. It is characterized histologically by the accumulation of cholesterol esters, cholesterol, and other neutral lipids [6], which when dissolved during histological preparation methods show a clear cytoplasm. RCC can also be considered a metabolic disease because metabolic pathways are strongly altered in RCC, including glycolysis, amino acid metabolism, and lipid metabolism [7,8]

Understanding the biology of ccRCC starts with the discovery and characterization of the *Vhl* gene. The loss or mutation of the *Vhl* gene, at the short arm of chromosome 3, is generally considered to be one of the obligate initiating steps in the development of ccRCC [9]. Germline mutations of the *Vhl* gene cause autosomal dominant hereditary von Hippel –Lindau familial cancer syndrome characterized by an increased risk of tumor development in multiple organs, including the kidney [10]. Associated focal lesions, such as ccRCC, arise from the inactivation or silencing of the remaining normal (wild-type) *Vhl* allele. Remarkably, biallelic *Vhl* mutations or, less frequently, hypermethylation are very common in sporadic ccRCC, meaning that the *Vhl* gene behaves like a classical Knudson two-hit tumor suppressor gene. The main function of the *Vhl* gene product, pVHL, is to regulate the cell’s response to oxygen availability. It functions as a subunit of the E3 ubiquitin ligase complex, which mediates the proteasomal degradation of an oxygen-dependent transcription factor called hypoxia inducible factor (HIFα). HIFα exists as three isoforms, HIF-1α, HIF-2α, and HIF-3α, with the HIF-2α isoform being most directly associated with ccRCC carcinogenesis. Under hypoxic conditions, HIF-2α heterodimerizes with an aryl hydrocarbon receptor nuclear translocator (ARNT, also known as HIF-1β) to form an active transcription factor complex that upregulates the expression of hypoxia-inducible genes, such as vascular endothelial growth factor (VEGF) and erythropoietin (Epo), to counteract hypoxia and increase tissue oxygenation [11]. Under normal conditions, oxygen-dependent post-translational modifications on HIF-2α allow pVHL to recognize and target HIF-2α for rapid degradation. In RCC, the loss of pVHL thus mimics hypoxia and leads to excess HIF activity and the subsequent activation of the transcription of hundreds of HIF target genes that participate in angiogenesis, cell migration, epithelial–mesenchymal transition (EMT), extracellular matrix remodeling, glucose and lipid metabolism, immune evasion, and metastasis [12]. An important gene is the one encoding for VEGF which is a major driver of angiogenesis and thereby supplies the tumor with more nutrients and oxygen to accelerate its growth and progression. Drugs that inhibit VEGF production or its interaction with VEGF receptors have become a central approach of ccRCC treatment [13,14].

To date, approved standard therapies for renal cancer mainly focus on targeting neoangiogenesis and the immune system. This is conducted either by monotherapies or by combination therapies with VEGFR tyrosine kinase inhibitors, checkpoint inhibitors, or mTOR inhibitors such as everolimus. However, these therapies have multiple severe adverse effects including an increased risk for infections and the development of drug resistance [15]. Therefore, novel targets are needed which, when blocked by combination therapy, results in synergistic anti-cancer effects and thus allow a reduction of the drug dose.

Preclinical data also indicate that HIF-2α antagonists, which block HIF pathway activation and therefore inhibit HIF-2α target gene activation, can inhibit tumor growth in ccRCC [16].

Since HIF-1/2α are regulating a multitude of genes [12,17], including lipid-regulating enzymes, and HIF-1/2α are themselves regulated by lipids, including prostaglandins and sphingolipids; this suggests that lipids may take center stage in renal cancer development, or could represent new targets for an intervention therapy.

## 2. Sphingolipids in Renal Cancer

Sphingolipids represent a vast class of lipids characterized by the presence of a sphingoid backbone in their structure. They are important constituents of cellular membranes, but are increasingly acknowledged for their role as signaling molecules.

**Figure 1:**
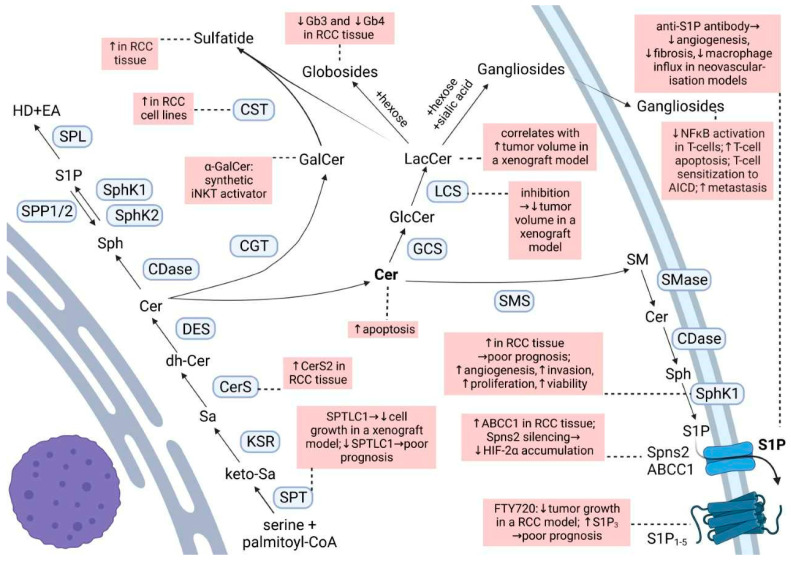
Sphingolipid biosynthesis and degradation routes. Changes reported for RCC are highlighted in pink boxes. For abbreviations, see text.

Ceramide is the main hub of the sphingolipid pathways (Figure 1). It is produced either through the de novo synthetic pathway, the salvage pathway, or the hydrolytic pathway. Ceramide may then be phosphorylated to form ceramide 1-phosphate (C1P), deacylated to sphingosine, or condensed with phosphatidylcholine to give sphingomyelin or glucose/galactose to give cerebrosides [18,19]. Ceramide is known as a pro-apoptotic molecule, and many commonly used chemo-therapeutic agents induce cancer cell apoptosis by activating the acid sphingomyelinase and increasing ceramide formation [20]. Additionally, in RCC cells, exposure to exogenous C6-ceramide, or increasing endogenous ceramide by a ceramidase inhibitor, had a cytotoxic effect [21].

Phosphorylation of sphingosine by the two sphingosine kinases (SphK1 and SphK2) yields sphingosine 1-phosphate (S1P), which is a potent bioactive lipid involved in processes such as proliferation, migration, angiogenesis, lymphocyte trafficking, and endothelial permeability [18,22]. Although a few intracellular targets of S1P are described, S1P is mostly known for its autocrine and paracrine functions through the five G protein-coupled receptors called S1P_1–5_. Due to the anti-apoptotic and pro-angiogenic roles of S1P, the SphK/S1P/S1PR axis attracts special interest in cancer treatment [23]. More specifically, this axis seems to be a master regulator of hypoxia by regulating HIF-1α and HIF-2α protein levels in human cancer cell lines including VHL-deficient ccRCC [24].

In this regard, SphK1 inhibition decreases HIF-1α levels by stimulating its degradation in a pVHL-dependent manner. When pVHL is deficient as in the RCC10 cell line, HIF-1α levels are constitutively high and cannot be influenced by hypoxia or SphK1 inhibition [25]. Moreover, SphK1 activity also controls HIF-2α expression and transcriptional activity, as SphK1 silencing promotes a VHL-independent HIF-2α loss which results in reduced cell proliferation in ccRCC [26].

Interestingly, HIF-2α is also capable of regulating SphK1 levels by stimulating its gene transcription, protein expression, and enzyme activity. This is followed by increased intracellular S1P production, S1P release, and S1P receptor activation [27]. According to The Cancer Genome Atlas (TCGA) RNA seq database [28], SphK1 expression is 2.7-fold higher in solid tumor tissue from ccRCC patients, and S1P is increased in RCC tissue compared to healthy tissue [29]. This is associated with poor survival and contributes to the resistance to the multi-kinase VEGFR inhibitor sunitinib [28,30]. Mechanistically, it might involve the increased invasion mediated by S1P_2_-dependent FAK phosphorylation [28], increased viability, and proliferation through Akt/mTOR [30], as well as an S1P_1+3_-mediated increase in angiogenesis [28]. Thus, the SphK1/S1P/S1PR axis is involved both in autocrine signaling to promote tumor growth, as well as in paracrine signaling to augment angiogenesis. Consequently, SphK1 inhibition was suggested as a possible strategy to control tumor hypoxia and its consequences [31].

In order to prevent the stimulation of S1PRs and downstream signaling, antibodies against S1P [32] or an extracellularly acting recombinant S1P lyase from *Symbiobacterium thermophilum* (stSPL) [33] were developed. Using the CAM model, Huwiler et al. (2011) demonstrated that this stSPL can indeed reduce tumor cell-induced angiogenesis. However, the short half-life in vivo, resulting in the rapid recovery of plasma S1P upon intravenous injection of stSPL in mice, hindered its further development.

Based on highly promising data of monoclonal murine (LT1002, Sphingomab) and humanized (LT1009, Sonepcizumab) S1P-specific antibodies in various preclinical models (cancer cell lines and in the retinal and choroidal neovascularization models in mice) [34,35,36], clinical studies were initiated. Two formulations of sonepcizumab were designed, one for an intravitreous application (iSONEP) to treat exudative age-related macular degeneration and one for an intravenous infusion (ASONEP) to treat metastatic RCC. Despite the good safety and tolerability profile, sonepcizumab did not reach the primary endpoint of progression-free survival and the study with refractory RCC was terminated [37]. Several limitations must be noted, such as the small patient number and a median of two prior failed therapies against VEGF/VEGFR, mTOR, or immunotherapy, which might anticipate a shorter progression-free survival [37]. On the other hand, the encouraging overall survival (>20 months in a heavily pretreated population) and the favorable safety profile of sonepcizumab suggested that this agent could be explored in combination with the currently approved agents for metastatic RCC [37]. So far, no such clinical trials were initiated.

Fingolimod (FTY720) which is an approved immunomodulator for the treatment of multiple sclerosis, acts as an unspecific agonist of all S1P receptors, except S1P_2_, and as a functional antagonist of S1P_1_ [38]. In several HIF-2α-resistant ccRCC cell lines, as well as in HIF-2α-resistant mouse ccRCC models, FTY720 showed anti-proliferative and anti-tumor effects [24]. Whether the modulation of all S1PRs or only one of them is needed for these actions is not well understood. Fischl et al. (2019) recently reported that S1P_1_ antagonism is sufficient to enhance the efficacy of the VEGFR inhibitor sunitinib in vitro and in vivo in the postnatal retinal angiogenesis model and in the RCC murine tumor model [39]. This combination not only disrupted the tumor vascular beds, but also decreased the tumor volume and increased tumor cell death compared with monotherapies [39].

On the other hand, siponimod, which is an S1P_1+5_ modulator, was devoid of anti-proliferative effects in RCC colony formation assays, which was attributed to the inability of siponimod to target the S1P_3_ receptor [24]. Notably, RNA sequencing of five human ccRCC cell lines (769-P, A498, 786-O, SLR22, and RCC4) revealed that different cell lines expressed different levels of the five genes, with S1P_1_ and S1P_3_ being the most abundantly expressed subtypes [24]. In RCC, the increased expression of insulin-like growth factor 2 mRNA-binding proteins enhances the stability of S1P_3_ mRNA promoting cell proliferation and migration [40], while patients with RCC characterized by a high expression of S1P_3_ have significantly worse overall survival [41]. These findings highlight the importance of a more selective approach when targeting the S1P receptors and more specifically of the potential of S1P_3_ antagonists in RCC treatment.

The S1P_2_ also deserves special attention, as this receptor subtype mediates an upregulation of connective tissue growth factor (CTGF) in the human cRCC cell line A498 following d16:1 S1P stimulation [42]. In this study, it was reported that d16:1 S1P modulates conventional d18:1 S1P signaling by acting as a more potent agonist at the S1P_2_ than the d18:1 S1P. It must be noted that all sphingoid bases, including d16, d18, and d20 chains, are produced in a rate-limiting step catalyzed by the same enzyme, the serine palmitoyltransferase (SPT) [43].

Recently, it was reported that the decreased expression of one of the two major subunits of SPT, SPTLC1, predicts a poorer outcome in ccRCC patients and is significantly associated with disease progression [44]. Moreover, SPTLC1 was decreased in RCC tissues compared to non-tumor tissues. The forced expression of SPTLC1 could significantly inhibit cell growth in vitro and in vivo in a nude mice xenograft RCC model via, at least in part, modulating Akt/FOXO1 signaling pathway [45].

Interestingly, although the SPTLC1 mRNA levels decrease with the increase of the ccRCC stage [44], which might suggest a reduced production of sphingoid bases, the content of dihydrosphingosine increased progressively with the increasing malignancy grade [29]. Moreover, the level of dihydroceramide, which is an immediate precursor of ceramide, was elevated in G4 tumors, but not in lower malignancy grades [29].

On the contrary, ceramide content, which is at a higher level in ccRCC than in non-cancerous kidney tissues, remained stable in tumors of higher malignancy grades despite the accumulation of dihydrosphingosine and dihydroceramide [29]. This indicates either a block in the ceramide synthesis or a shift towards a particular ceramide subspecies. Notably, mammals have six ceramide synthases (CerS1-6), each exhibiting a preference for the chain length of the fatty acyl-CoA substrate and producing a distinct ceramide species [46]. Indeed, data from RNA-seq databases show that RCC tumors exhibit increased CerS2 mRNA, which is inversely correlated with CerS6 mRNA in ABCB1+ clear cell carcinomas compared to normal tissue [47]. Lipidomics analysis also showed a shift to predominantly longer chain ceramide and sphingomyelin species in chemoresistant ABCB1^high^ cells [47].

The abovementioned study by Młynarczyk et al. (2022) [29] reported on the expression levels of the major S1P-degrading or exporting factors, i.e., SPL, SPP1, SPP2, Spns2, and ABCC1, but no clear trend for a malignancy grade-depended expression was detected. Interestingly, the silencing of Spns2 blocked HIF-2α accumulation in ccRCC cell lines, thus mimicking the effect of the anti-S1P antibody [26] and again highlighting the importance of S1P signaling in the regulation of HIF-2α as a driver of a more aggressive disease in RCC.

## 3. Glycosphingolipids in Renal Cancer

Galactosylceramide, or galactocerebroside, is produced from ceramide by the attachment of a galactose residue at the 1-hydroxyl moiety. α-Galactosylceramide (KRN-7000, α-GalCer) is a synthetic glycosphingolipid which acts as a synthetic iNKT (invariant Natural Killer T) cell ligand when presented by CD1d [48]. This interaction activates the iNKT and increases the number of iNKT and the production of pro-inflammatory cytokines which later activate the NK, tumor-specific, CD4+, CD8+ T cells, and B cells [49]. Numerous clinical trials have demonstrated tumor regression, a stable disease, partial response, or increased median survival time with α-GalCer therapy in various cancers; however, studies in RCC are missing [49]. Although this immunotherapeutic vaccine approach was suggested to be of benefit in RCC [48], the efficacy is unclear due to contradictory results and scarce studies. In this regard, in vitro α-GalCer-loaded dendritic cells induced the proliferation of iNKT cells derived from a pediatric papillary RCC [50]. However, NK T cells isolated from peripheral blood mononuclear cells (PBMCs) of a fraction of patients with metastatic (m)RCC showed no functional activity towards autologous tumor cells in the presence of α-GalCer [51].

Galactosylceramide is used by cerebroside/galactosylceramide–sulfotransferase (CST) to produce sulfatide. An elevated expression of sulfatide is commonly found in many human cancer cell lines and tissues and may possibly be used as a biomarker of some cancer cells [52]. Sulfatide is a major L-selectin ligand in the kidney, and the binding between L-selectin and sulfatide plays an essential role in monocyte infiltration into the kidney interstitium [52]. In various RCC cell lines, a marked increase of CST mRNA and activity was observed [52]. Moreover, lactosyl- and galactosylceramide sulfate are markedly increased in RCC as compared to healthy tissue, accompanied by significantly elevated activities of their respective sulfotransferases [53,54]. This is also reflected in the plasma and urine of RCC patients, where elevated concentrations of lactosylsulfatides were stage-dependent and more emphasized in late-stage RCC [55]. Nevertheless, Porubsky et al. (2021) could not confirm an association between CST expression and malignant clinical behavior of RCC [56]. Thus, the role of sulfoglycosphingolipids in RCC beyond the potential role as biomarkers for early RCC diagnosis [57] at this moment lacks evidence.

The glucosylceramide synthase (GCS) is overexpressed in metastatic breast carcinoma [58] and drug-resistant breast, ovary, cervical, and colon cancer cells [59]. GCS upregulation is also part of the genetic signature for the progression and metastasis of RCC based on the results of gene-expression profiling of human RCC tumor samples [60]. Since an overexpression of GCS confers drug resistance and the suppression of GCS expression overcomes the resistance by enhancing drug uptake and ceramide-induced apoptosis in breast cancer cells [58,61], this suggests a mechanism that should also be considered in RCC.

Glucosylceramide serves as a substrate for the lactosylceramide synthase to build lactosylceramide. In a xenograft mouse model of RCC a significant correlation between the increase in the mass of lactosylceramide and the tumor volume was detected, and inhibition of GCS and lactosyl-ceramide synthase activities led to a decrease in tumor volume [62].

Starting from lactosylceramide, globosides can be formed by the attachment of sugar residues, and gangliosides by the attachment of sugar residues and sialic acid.

It is now generally accepted that gangliosides produced by cancer cells play a role in immune escape. In the context of RCC, it was demonstrated that explanted RCC tumors produce soluble gangliosides that inhibit the nuclear factor κB activation of co-cultured T cells [63], sensitize T cells to activation-induced cell death [64], or directly induce T-cell apoptosis by caspase activation [65].

For instance, RCC patients present with increased apoptotic T cells compared with T cells from healthy donors, and the majority of those apoptotic T cells were GM2(+) which they acquired from tumor shedding [66]. GM2 originating from RCC was also shown to promote T cell dysfunction by down-regulating cytokine production [67]. Not only do RCCs display increased levels of the gangliosides GD1a, GM1, and GM2 as compared with cells of the normal kidney [65,68], but they also synthesize disialogangliosides which seemingly promote the metastatic capabilities through a mechanism involving the formation of microembolisms [69].

Disialosyl globopentaosylceramide (DSGb5) is a dominant ganglioside isolated from RCC tissues [70] which binds to sialic acid-binding Ig-like lectin-7 (Siglec-7) expressed on natural killer (NK) cells, thereby inhibiting NK-cell cytotoxicity [71]. Higher DSGb5 expression exhibits greater migration potential in ACHN RCC cells and is correlated with metastasis in RCC patients [71,72]. Other gangliosides, such as GalNAc disialosyl lactotetraosylceramide [73] and monosialosyl galactosylgloboside (MSGG) [74,75], bring a higher risk of metastasis; however, the exact mechanisms are still not thoroughly investigated.

Unlike gangliosides, the globosides globotriaosylceramide (Gb3) and globotetraosylceramide (Gb4) are markedly reduced in ccRCC tissue as compared to healthy renal tissue, and they decrease progressively with increasing malignancy grade [29].

There seems to be a connection between the ganglioside and globoside content in RCC cells driven by the action of the plasma membrane sialidase NEU3 [76]. NEU3 silencing in a human primary RCC cell line led to an increase in ganglioside content (e.g., GD1a, GM2, and GM3), and a decrease in the globoside Gb3 content [76]. Moreover, the production of ganglio-series gangliosides was enhanced to the detriment of globo-series gangliosides, particularly MSGG [76]. The effects of this silencing on RCC cell malignant phenotype and behavior were significant and involved drug resistance, invasive potential, and adhesion [76]. Nevertheless, other mechanisms could still play a role in these findings, as an increase of GM3 simultaneous with a decrease of MSGG in the human RCC cell line ACHN following brefeldin A treatment was marked by growth suppression and correlated to the pattern observed in RCC cases with a more favorable prognosis [77].

Considering the proposed functions of gangliosides in other tumors, such as binding to endothelial cells through carbohydrate–carbohydrate interactions, modulation of adhesion receptors, or the promotion of tumor-associated angiogenesis [69], this opens new avenues of research in the roles of gangliosides in RCC progression. Altogether, gangliosides expressed on RCC tumors may be important markers of tumor progression and target antigens for immunotherapy.

## 4. Free Fatty Acids in Renal Cancer

### 4.1. Exogenous Uptake of Fatty Acids

**Figure 2:**
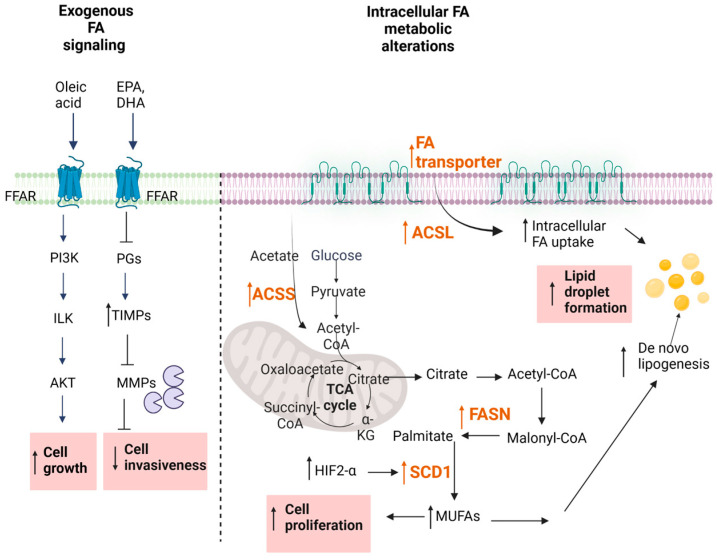
Fatty acid metabolism. Changes reported for RCC are highlighted in pink boxes. For abbreviations, see text.

Over the last decades, extensive studies have approached the effect of free fatty acids (FA), particularly of ω3-polyunsaturated fatty acids (PUFAs) on cancer cells, and many epidemiological studies support the idea of a correlation between dietary FA intake and the development of cancer [78]. Traditionally, saturated FAs have long been considered harmful, whereas plant monounsaturated FAs (MUFAs) such as oleic acid and ω-3 PUFAs were associated with a lower cancer mortality (Figure 2). However, systematic reviews reveal only weak epidemiological evidence for a clear protection by MUFAs and ω3-PUFAs [79,80]. Certain studies even concluded that certain MUFAs and PUFAs can promote cancer development [81,82,83,84,85].

Data approaching specifically the effect of FA intake on RCC is also scarce and contradictory. In an in vitro study in an RCC cell line, it was shown that PUFAs, including docosahexaenoic acid (DHA) and eicosapentaenoic acid (EPA), were reducing the invasive profile of cells by upregulating the tissue inhibitor of metalloproteinase (TIMP)-1 [86]. It was hypothesized that ω-3 PUFAs modulated TIMP-1 synthesis by competing with the ω6-PUFA arachidonic acid for cyclo-oxygenase activity. This was supported by detecting reduced prostaglandin E_2_ (PGE_2_) production upon the addition of exogenous DHA which in turn elevated TIMP-1 protein levels. In a case control study conducted in Italy, both PUFAs and MUFAs seemed to be protective [87], while a study evaluating the situation in a U.S. population cohort reported an elevated RCC risk with the increased dietary intake of animal fat, saturated fat, oleic acid, and cholesterol [88]. Furthermore, a pooled analysis of 13 prospective studies showed statistically significant positive associations in pooled age-adjusted models for intakes of total fat, saturated fat, monounsaturated fat, polyunsaturated fat, and cholesterol and the incidence of RCC. However, after adjusting for BMI, fruit and vegetable intake, and alcohol intake, the statistically significant association was no longer seen [89]. Clearly more studies are needed to prove dietary FA intake as a risk factor for RCC development.

### 4.2. Regulation of Fatty Acid Signaling in Cancer

The regulation of oncogenic signaling by FAs has also been considered as a novel therapeutic approach in RCC. Depending on the chain length, FAs can either freely enter the cell or use transport proteins [90,91] (Figure 2). Alternatively, cell surface FA receptors exist, denoted FFARs [92,93] which are subdivided into two groups, the long-chain FFARs (FFAR1/GPR40 and FFAR4/GPR120) and the short-chain FFARs (FFAR2/GPR43 and FFAR3/GPR41). All these receptors belong to the superfamily of GPCRs. Their involvement in cancer cell growth and progression are only now beginning to be unmasked, but it seems that the long-chain FFARs have a different role than short-chain FFARs [93]. Various in vitro studies in prostate, breast, ovarian, and colon cancer cells reveal that dual FFAR1/FFAR4 agonists can reduce the proliferation and migration of cancer cells, supporting the usefulness of these receptors as pharmacological targets [94,95,96,97]. However, so far, no reports are available for FFAR involvement in RCC. Clearly, it will be important to optimize such FFAR agonists for selectivity and potency when considering them for further development.

### 4.3. Altered De Novo FA Synthesis

Metabolic reprogramming occurs because of mutations in cancer genes and alterations in cellular signaling. In addition to alterations in glucose and glutamine metabolism, increased de novo FA synthesis, uptake, and the suppression of FA oxidation, which eventually leads to lipid droplet (LD) formation, have been recently shown to be a hallmark of the disordered intermediary metabolism in cancer cells [98,99].

The fatty-acid synthase (FASN) is the key metabolic multi-enzyme that is responsible for the terminal catalytic step in FA synthesis (Figure 2). FASN is present at high levels in most human malignancies, especially in gynecological, prostate, and colon cancers [100,101,102], and it is correlates with a worse prognosis [103]. Therefore, FASN is speculated to be a new therapeutic target in RCC.

In a first study, Horiguchi et al. showed increased FASN protein staining in immunohistochemical sections of RCC patients [104]. Positive FASN protein expression was associated with increased tumor aggressiveness and was an independent predictor of shortened cancer-specific survival, suggesting that FASN could be a predictive indicator of disease prognosis [104]. These data were later confirmed by another study [105], which assessed the differential mRNA expression of FASN in 533 ccRCC samples and 72 adjacent normal samples from a TCGA cohort. The data showed significantly increased FASN mRNA in ccRCC samples when compared to normal samples, and the elevated FASN mRNA correlated with a poor prognosis and malignant biological behaviors of ccRCC [105]. Similar data were also obtained by Yuan et al. [106] by using Western blot analysis and immunohistochemical staining of RCC tissue sections for FASN.

FASN up-regulation and its association with a poor prognosis holds true for other cancer types as well [107,108,109], making this a universal cancer feature and thus supporting its usefulness as a therapeutic target of ccRCC. Wettersten et al. [110] revealed that in RCC, metabolic reprogramming is grade-dependent. Interestingly, they reported that the levels of shorter chain FFAs (6:0, 8:0, 9:0, 10:0, and 12:0) were decreased in a tumor grade-dependent manner and this was probably due to an increase in their utilization. Nevertheless, also in this study, FASN was found to be increased on a protein level in cancer tissue when compared to the adjacent nontumor tissue [111]. A functional analysis of FASN in human ccRCC cells showed that down-regulation or overexpression of FASN significantly regulates ccRCC cell proliferation and migration by regulating EMT. Moreover, FASN inhibition also increased the apoptotic rate, decreased lipid droplet formation, and suppressed the mRNA expression of hub genes in EMT [105]. On top of this, the pharmacological inhibition of FASN reduced the growth and invasiveness of renal cancer cells in vitro and in vivo. One possible mechanism could be the disturbance of cell membrane functioning by down-regulated Her2 and EGFR and downstream STAT3 signaling [104], which was shown to play an important role in pancreatic cancer metastasis [112]. The ability of FASN inhibition to suppress cancer cell growth was also proven in a cell line of a pediatric malignant rhabdoid kidney tumor [113]. Additionally, a proteomic analysis of tissue samples of a Wilms tumor confirmed that the expression of FASN was significantly increased in the tumor tissues as compared to adjacent tissues and this was associated with a poorer prognosis [114,115]. All these data suggest that FASN plays a key role in ccRCC carcinogenesis and that the FASN expression level could be equally used as a predictor of poor prognosis in both pediatric and adult renal tumors.

Other than the *de novo* synthesis, other enzymes in the FA pathway are involved in the altered lipid metabolism of RCC such as altered FA activation, FA uptake, and the suppression of FA oxidation [99]. Once synthesized, FAs need to be activated by conversion to FA acyl-CoA esters by the action of acyl-CoA synthases (ACS) before they are further processed. Depending on the chain length of the Fas, ACSs are divided into different classes, comprising the very long chain (ACSVL), long chain (ACSL), medium chain (ACSM), and short chain (ACSS) synthases. Thus, ACSL converts FAs of C8-C22 chain lengths into an activated form. It represents a group of five isoforms, denoted ACSL1,3,4,5,6 [116]. Increased levels of all these enzymes have been suggested to be involved in molecular processes driving cancer growth and progression [116,117]. While a clear view on the relevance of all the ACSL isoforms in RCC is not yet available, it seems that at least ACSL3 could serve as a potential prognostic biomarker for immune infiltration in ccRCC [118]. Furthermore, ccRCC cells in vitro depend on ACSL3 for lipid droplet formation. Selective pharmacological inhibition or the genetic suppression of ACSL3 is cytotoxic for RCC cells and also reduces the tumor size in an orthotopic mouse cancer model [119]. These data propose that ACSL3 could indeed be a possible pharmacological target for RCC therapy.

The introduction of a double bond into the two saturated FAs palmitoyl-CoA and stearoyl-CoA by the action of the ∆9-stearoyl-CoA desaturase (SCD1) yields the monounsaturated FAs (MUFAs) palmitoleoyl-CoA and oleoyl-CoA which generally represent the main components of cellular lipids including phospholipids, triglycerides, and cholesterol esters.

Notably, in many cancer tissues, including RCC, SCD1 expression is up-regulated [120,121], and consistent with this observation, analyses of the FA profile in the serum of patients with different cancer types showed increased levels of MUFAs, and consequently a reduced ratio between SFA/MUFA. This ratio may also serve as a predictive marker of cancer aggressiveness and patient prognosis, but this would need to be validated in bigger cohorts of patients for the different cancer types [122,123,124,125]. In addition, SCD1 could also serve as a novel pharmacological cancer target. Recently, selective SCD1 inhibitors were developed and tested in disease models [125]. Among these inhibitors, A939572 proved efficient to reduce ccRCC cell growth in vitro, and in an in vivo mouse model, the combination of A939572 plus the mTOR inhibitor (temsirolimus) exerted a synergistic effect on tumor size reduction [121].

Interestingly, SCD1 is a hypoxia-regulated gene and involves HIF-2α as the key transcription factor. Furthermore, there seems to be a synergistic effect between HIF-2α and SCD1 on modulating RCC tumorigenic cell responses [120]. In view of the known regulation of HIF-2α by other lipid classes including sphingolipids (see Chapter 2), it is tempting to speculate that on the level of SCD1, a regulatory cross-talk between FA metabolism and other bioactive lipids also exists.

## 5. Eicosanoids in Renal Cancer

**Figure 3:**
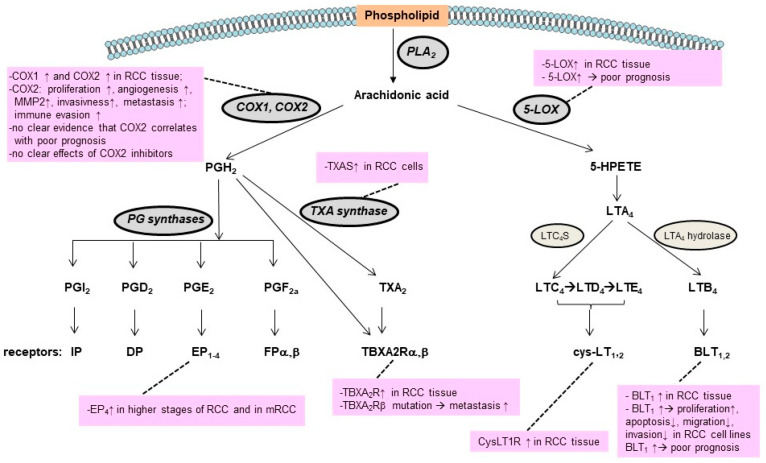
Prostaglandins and leukotrienes synthesis pathways and receptors involved. Changes reported for RCC are highlighted in pink boxes. For abbreviations, see text.

Eicosanoids are oxidized derivatives of 20-carbon PUFAs formed by the cyclooxygenase (COX), lipoxygenase (LOX), and cytochrome P450 (cytP450) enzymes (Figure 3). Arachidonic acid is the usual substrate for eicosanoid synthesis [126]. The COX pathways form prostaglandins (PGs) and thromboxanes (TXs), the LOX pathways form leukotrienes (LTs) and lipoxins (LXs), and the cytP450 pathways form various epoxy, hydroxy and dihydroxy derivatives [126].

Eicosanoids can modulate multiple biological processes including cell proliferation, adhesion, migration, angiogenesis, vascular permeability, and inflammatory responses [127]. An altered metabolism of arachidonic acid is a common feature of several epithelial-derived malignancies and has been shown to have crucial roles in cancer progression [128].

### 5.1. Prostaglandins

The COX/PGE_2_ pathway and the PGE_2_ receptors, denoted EP_1_, EP_2_, EP_3_, or EP_4_, have attracted special interest as pharmacological targets for RCC. These receptors belong to the GPCR family and couple to various G-proteins and downstream signaling cascades [129,130].

In renal physiology, COX-1 is involved in hemodynamic regulation, while COX-2 expression is regulated in response to intravascular volume and is important in maintaining salt and water homoeostasis, and both enzymes largely involve PGE_2_ [126]. COX-1 is expressed constitutively in the kidney, and while COX-2 is inducible in most tissues in response to injury or inflammation, COX-2 mRNA and protein are present at detectable levels in normal adult mammalian kidneys [131].

COX-2 expression seems to play a role in the inflammation–carcinoma sequence in various epithelial cancers including RCC [132]. The expression of both COX-1 and COX-2 correlates with the clinicopathological features of RCC, including tumor size, tumor stage, and tumor grade [132,133,134]. Yet, there is no clear relationship between COX-2 expression and patient survival [132], and the increased expression of COX-2 may not be an important prognostic factor in conventional RCC [135]. One study even found that COX-2 protein expression is associated with the slow development of metastases, and a favorable prognosis in metastatic RCC [136].

Despite these ambiguities, it is generally accepted that COX-2 exerts a pleiotropic and multifaceted role in carcinogenesis and cancer cell resistance to chemo- and radiotherapy [137]. The exact mechanisms of COX-2 contribution to RCC are under investigation but involve increased cell proliferation, angiogenesis, matrix metalloproteinase (MMP)-2 expression, invasiveness, and metastasis [134,138].

COX-2 in cancer also contributes to immune evasion through several mechanisms [139]. In the context of RCC, it was demonstrated that the overexpression of COX-2 in OS-RC-2 cells leads to higher PGE_2_ secretion which increases the percentage of T_regs_ in the CD4+Foxp3- T cells when cultured with medium supernatants [140]. These T_regs_ in turn suppress the proliferation of CD4+CD25- T cells and it is believed that this suppresses antitumor immunity [140]. Indeed, increased peritumoral T_regs_ predict a poor prognosis in ccRCC and are positively correlated with intratumoral COX-2 expression [141]. Another proposed mechanism is that RCC inhibits the host antitumor immune response by promoting PGE_2_ production by PBMC and a shift of the cytokine profile in favor of a Th2 response [142], or that RCC cells induce PGE_2_, IL-10, and TNF-α production by monocytes, which down-regulate the expression of the cell surface molecules involved in antigen presentation, as well as their endocytic capacity [143].

Acetylsalicylic acid (ASA) and non-ASA non-steroid anti-inflammatory drugs (NSAIDs) which act as unselective inhibitors of COX-1 and COX-2 have an anti-tumorigenic effect in several cancers. However, a meta-analysis found that non-ASA NSAIDs were associated with a higher incidence of RCC [144]. The association was stronger when non-ASA NSAIDs were used at higher doses and for longer periods of time [144]. Hamieh et al. (2018) further showed that in mRCC, there is no difference in the survival outcomes of ASA and non-ASA NSAIDs users compared to non-users [144]. Due to the unselective effect on COX-1 and COX-2, which causes gastrointestinal injury and nephropathy as side effects, more research is focused on COX-2 selective inhibitors [145].

COX-2 selective inhibitors show controversial results in vitro, and while some show cytotoxic effects [146], others do not induce a reduction of cell viability or proliferation of RCC cell lines [147]. In cRCC xenograft models, the COX-2 inhibitor celecoxib inhibited tumor growth only in one out of four different models [148]. Patient studies are also controversial. While a phase-II clinical trial with patients with mRCC demonstrated excellent efficacy of a combination treatment with the COX-2 inhibitor meloxicam and IFN-α [149], the selective COX-2 inhibitor celecoxib in combination with IFN-α did not increase the objective response rate or time to disease progression in mRCC [150]. Additionally, in mRCC patients with maximal COX-2 staining by immunohistochemistry, the celecoxib plus IFN-α combination did not significantly enhance overall response rates over IFNα monotherapy [151].

A combination of COX-2 inhibitors with other drugs, except INF-α, might be a better approach. Notably, a combination treatment comprising meloxicam, cimetidine and a renin–angiotensin system (RAS) inhibitor in mRCC produced favorable responses in a phase II clinical trial of advanced RCC [152]. As COX-2 expression is increased in the hypoxic areas of cRCC xenografts [148], and the activation of the COX-2/PGE_2_ pathway in RCC cells was hypothesized to lead to the development of sunitinib resistance [153], a concurrent therapy with celecoxib and sunitinib delayed the time to progression in an RCC xenograft model [148] which prompts clinical investigation.

COX-2 expression in RCC tissue does not seem to be correlated to invasion or metastasis [132,134]. Still, in vitro and in vivo data show an association of a COX-2/PGE_2_/EP axis with invasion and metastasis. This is because in addition to the overproduction of PGE_2_, the aberrant expression of its receptors can also amplify PGE_2_ signaling, which can facilitate cancer promotion and metastasis [138]. In particular, EP_2_ and EP_4_ may play important roles in the malignant behavior of RCC. EP_2_ expression did not differ between normal and RCC tissues; however, it was significantly higher in metastasized tumors than in tumors without metastasis. EP_4_ is closely associated with pathological features, and the stage and metastasis of RCC, thus is a significant predictor of survival in RCC patients [154]. Experiments with RCC cells also evidenced increased EP_4_ as compared to normal tubular epithelial cells [155], and a reduced tumor intravasation when EP_4_ was down-regulated in a xenograft model [156]. Moreover, in a CAM assay, the inhibition of EP_4_ attenuated RCC intravasation and metastasis by downregulating CD24, a ligand to the adhesion molecule P-selectin [156]. In another study, PGE_2_ promoted RCC7 cell invasion through EP_4_ and small GTPase Rap signaling [154]. The same group later showed that PGE_2_ increased SN12C cell invasion through a signaling pathway that encompasses EP_2_ and EP_4_, Akt, small GTPase RalA, and Ral GTP inactivator RGC2, further dissecting the downstream pathway [157]. Another proposed mechanism includes a decrease in E-cadherin expression, and an increase of CD44 expression and adhesion to hyaluronan in RCC cells with forced COX-2 expression compared with parental cells [158]. All this, together with the known role of EP_4_ in the formation of an immunosuppressive tumor microenvironment [159,160], highlights the therapeutic value of targeting EP_4_ in RCC. So far, two EP_4_ antagonists, E7046 and BMS-986310, have been explored in clinical studies for advanced RCC (NCT02540291, NCT03661632). E7046 showed no dose-limiting toxicities but induced changes in genes downstream of EP_4_ [159], proposing further development in this field.

### 5.2. Thromboxane

In the COX pathway, PGH_2_, the immediate metabolite from arachidonic acid, can be further metabolized to thromboxane A_2_ (TXA_2_) by the enzyme TXA_2_ synthase (Figure 3). The short-lived TXA_2_ can then act on the thromboxane receptor (TBXA_2_R, TP) which exists in two isoforms (α and β). Although TXA_2_ appears to be of minor importance in the maintenance of renal functioning under physiological circumstances [161], an overexpression of TXA_2_S mRNA has been reported in renal cancer cells [162]. The relevance of this observation for RCC remains open.

TBXA_2_R is up-regulated in various tumors, and data from the Cancer Cell Line Encyclopedia show significant up-regulation in renal cancer, although without specification of the type of renal cancer [162]. Interestingly, a mutation of the β isoform of TBXA_2_R (SNP: rs200445019) was associated with metastatic disease at multiple tissue sites originating from primary renal cancer [162]. Another study shows that the β isoform is highly expressed in RCC cell lines which suggests the TBXA_2_R-β a candidate for further exploration [163].

### 5.3. Leukotrienes

Emerging evidence suggests that the LOX pathways are also involved in carcinogenesis. In general, 5-LOX and 12-LOX have potential pro-carcinogenic roles, whereas 15-LOX-2 is thought to have an anti-carcinogenic effect, and the role of 15-LOX-1 remains controversial [128].

Based on the TCGA database, an increased expression of 5-LOX in ccRCC tumors is associated with decreased overall survival [119]. An analysis of primary ccRCC tissues revealed that in the majority of tissues, the protein levels of 5-LOX are significantly increased compared to normal renal cortex biopsies [164,165], which correlated with a large tumor size, but not with the tumor grade or vein invasion [164]. Moreover, 5-LOX was frequently overexpressed in pVHL-reduced and in VEGF-positive ccRCC tumors, which as discussed in the introduction, represent two frequent alterations in ccRCC [164]. Cell culture experiments with RCC cell lines also demonstrate that the loss of pVHL expression leads to high basal 5-LOX and VEGF expression, and that VEGF expression is strongly induced by 5-LOX metabolites in RCC cell lines [164]. Moreover, 5-LOX was found to be a downstream regulator of the ACSL3-induced sensitization to ferroptosis, a non-apoptotic form of cell death, possibly through the production of lipid peroxides from PUFAs [119]. It is reported that the inhibition of 5-LOX in vitro causes a reduction of RCC cells in a concentration- and time-dependent manner [165]. Nevertheless, the concentrations needed to achieve a reduction of viability were high, ranging between 20–80 μM of caffeic acid [166]. Other novel 5-LOX inhibitors, such as the 2,5-dihydroxycinnamic acid phenethyl ester, might be a better alternative. Results showed that this ester compound induced apoptosis at an IC_50_ of 8 μM and possibly impaired the autophagic flux in the VHL-negative RCC4 cell line [167]. Although an FDA-approved 5-LOX inhibitor (zileuton) exists and is used in asthmatic patients [168], so far no study has addressed its efficacy in an RCC setting.

5-LOX oxidizes the arachidonic acid to 5-hydroperoxyeicosatetraenoic acid (5-HPETE), and the subsequent metabolism of 5-HPETE to 5-HETE and to the unstable LTA_4_. LTA_4_ can then be converted to LTB_4_, which binds to LTB_4_ receptors (LTB_4_R, also known as BLT_1,_ and LTB_4_R2, also known as BLT_2_), or to cysteinyl LTs (LTC_4_, LTD_4,_ and LTE_4_), which bind to the cysLT_1_ or cysLT_2_ receptors [128] (Figure 3).

A very recent study showed that LTB_4_R overexpression promoted proliferation and inhibited apoptosis of ccRCC cells by stimulating the AKT/mTOR signaling pathway [169]. Moreover, migration and invasion were inhibited when LTB_4_R was depleted [169]. According to the TCGA database, LTB_4_R was overexpressed in ccRCC samples compared to normal samples and patients with higher LTB_4_R expressions showed significantly poorer overall survival than patients with lower LTB_4_R [169]. Accordingly, LTB_4_R was identified as a prognostic biomarker for patients with ccRCC [170]. A recent study also identified the LTB_4_R2, a lower affinity LTB_4_ receptor, to be positively correlated with poor overall survival for patients with ccRCC, and interestingly a positive correlation between T_regs_ and T cell exhaustion marker genes with LTB_4_R2 was found [171]. Nevertheless, in vitro and in vivo studies to corroborate these findings are lacking.

The receptors to cysteinyl LTs have also attracted interest as the CysLT_1_R was shown to be significantly up-regulated in RCC tissues than in normal kidney tissues, and this expression was higher in high-grade compared to low-grade cancer [172,173]. A CysLT_1_R antagonist in vitro induced a reduction in RCC cell viability through early apoptosis; however, the effect was only evident at 100 µM [172,173].

A recent epidemiological study showed that asthmatic patients taking the CysLTR antagonists montelukast or zafirlukast had a lower risk of several types of cancers compared to non-users [174]. Regarding RCC, zafirlukast induces VHL- and HIF-2α-dependent oxidative cell death in ccRCC cells, which can be rescued with antioxidants and a PARP-1 inhibitor [175]. Furthermore, the inhibition of HIF-2α degradation sensitized wild-type pVHL-expressing cells towards zafirlukast-induced cell death, which suggested that HIF-2α activity might be an important determinant for zafirlukast-mediated cell death [175]. Besides these findings, evidence is still lacking about the potential of LT receptor antagonists in RCC.

12-LOX oxidizes arachidonic acid at position C-12 to produce 12-hydroperoxyeicosatetraenoic acid (12-HPETE) and then 12-HETE. While a 12-LOX expression level is slightly detected in normal kidney tissue, a marked expression is detected in RCC tissues [165]. Additionally, in the RCC cell lines Caki-1, A498, and RC-1, the expression of 12-LOX was confirmed on an mRNA level [176]. Furthermore, the 12-LOX inhibitor baicalein caused a growth inhibition of all three kinds of RCC cells in a concentration- and time-dependent manner [176].

Tumor-associated macrophages, which frequently infiltrate RCC, display enhanced 15-LOX2 activity and the secretion of its major product 15(S)-HETE [177]. This enhanced 15-LOX2/15(*S*)-HETE activity in the RCC tumor microenvironment positively affects the production of the proinflammatory chemokine CCL2 and immunosuppressive cytokine IL-10, thus promoting local immunosuppression and tumor evasion [177,178].

Overall, 5-, 12-, and 15-LOX-1 coexist in the human kidney but show opposite trends in the course of cancer progression, with increased 15-LOX-1 and decreased 5- and 12-LOX levels at the onset, then reversing with the progressing stage of the disease or grade of tumor [179]. Clearly, more research is needed in the lipoxygenase pathway with regards to RCC.

### 5.4. HETE and EETs

The role of HETEs and the epoxyeicosatrienoic acids (EETs) in cancer is rather neglected. However, some evidence shows that products of the cytP450 enzymes, notably 20-HETE, can play an important role in cell growth and cancer development [180]. Alexanian et al. (2009) reported that the 20-HETE-generating enzymes CYP4F2 and CYP4F3 are expressed on the mRNA level in RCC cells [181]. The inhibition of 20-HETE synthesis suppressed the proliferation of 786-O and 769-P cells in vitro, while the administration of an apparent 20-HETE signaling antagonist reduces tumor growth in an ectopic mouse model of ccRCC [181]. An LC/MS/MS analysis of the RCC cell line 786-O also revealed the presence of 15-, 12-, and 5-HETEs and 14-, 15-, 11-, 12-, 8-, and 9-EETs [181]. Their individual contribution to RCC is currently unknown.

## 6. Cannabinoids in Renal Cancer

Cannabinoids comprise a class of highly bioactive lipids that include the psychoactive components of the plant *Cannabis sativa*, i.e., ^9^Δ-tetrahydrocannabinol (THC) and cannabidiol, and the endogenously synthesized endocannabinoids. The two main endocannabinoid species are the arachidonic acid derivatives arachidonoylethanolamine (anandamide, AEA) and 2-arachidonoylglycerol (2-AG). All these compounds act as ligands of specific cell surface cannabinoid receptors, named CB_1_ and CB_2_, which are G protein-coupled receptors expressed in the central nervous system but also in the periphery.

The biosynthesis of AEA and 2-AG occurs by different parallel routes and involves several enzymes [182,183,184]. The main degrading enzyme of AEA is fatty acid amide hydrolase (FAAH), a ubiquitously expressed intracellular membrane-bound serine hydrolase, while 2-AG is mainly degraded by a monoacylglycerol lipase (MAGL) [184]. To understand the role of cannabinoids in physiological and pathophysiological processes, it is essential to take into account all these anabolic and catabolic enzymes, transporters, and receptors.

Many in vitro studies in different cancer cells and in mouse cancer models were performed to show the growth inhibitory and pro-apoptotic role of THC and endocannabinoids, as well as of selective CB_1_ and CB_2_ agonists [185]. Various mechanisms were suggested including an enhancement of ceramide formation [186], or an inhibition of EGF and IGF signaling [187], and even receptor-independent mechanisms [188], for example by targeting the vanilloid receptor TRPV1 [189].

In addition, the modulation of the degrading enzymes, FAAH for AEA, and MAGL for 2-AG, appears to play a role in cancer development [190]. In this view, it was shown that MAGL expression correlates with the malignancy degree in different types of carcinoma and that its enzymatic activity could promote cancer aggressiveness and metastasis formation and thus, that the inhibition of MAGL could have an anti-tumorigenic effect [191,192]. Similarly, FAAH inhibitors can reduce cancer cell growth in vitro and in vivo in mouse xenograft tumor models [193,194,195].

While CB_1_ and CB_2_ are obviously involved in anti-tumorigenic mechanisms, it is uncertain whether the expression levels of the two receptors can serve as prognostic markers and allow a conclusion on the malignancy degree of the cancer. Larrinaga et al. [196] analyzed the expression of both CB receptor subtypes in 20 surgically obtained tissue samples of ccRCC. They found a down-regulated CB_1_ expression in tumor tissue when compared to adjacent non-neoplastic tissue. They concluded that ccRCC is characterized by a reduced CB_1_ expression and an absent CB_2_ expression which is in line with the idea that reduced CB_1/2_ signaling promotes cancer cell growth.

In contrast, Wang et al. [197] reported on a positive expression not only of CB_1_, but also of CB_2_, in RCC tissue, and they observed an up-regulation of CB_2_ in RCC tissues as compared to non-tumoral adjacent tissues. Moreover, by down-regulating CB_2_ by siRNA in RCC cell lines, or using the CB_2_ inverse agonist AM630, the proliferation and migration of cells were inhibited. These data led them to propose that specifically CB_2_ presented an independent prognostic factor for the overall survival of RCC patients, and the use of CB_2_ inverse agonists or antagonists could serve as a novel pharmacological strategy in RCC [197]. When characterizing a series of eight different human RCC cell lines for CB_1_ and CB_2_ expression, it appears that both receptors are expressed, even that the expression of CB_2_ is higher than CB_1_ within the same cell line [198]. Notably, CB_2_ agonists, such as celastrol, have recently also been suggested as an anti-fibrotic drug in renal fibrosis [199,200] and therefore, it will be important to rule out a pro-fibrotic effect of CB_2_ antagonists before considering such drugs for RCC treatment.

In addition, a retrospective, observational study demonstrated a possible interaction between cannabis use and immunotherapy among cancer patients, including ccRCC, namely a decrease in response rate to immunotherapy when using cannabis. This observed effect could be explained by the immunosuppressive effects of cannabis [201,202], However, these results should be cautiously interpreted because of the study’s design limitations and the nonrepresentative population, given the high number of lung cancer patients (98 of 140) [203].

In summary, few studies have explored the in vitro effects of cannabinoids in RCC and even fewer studies provide population-based evidence for their effectiveness. Thus, further research is required not only to evaluate the crosstalk between cancer signaling pathways and the endocannabinoid system, but also large randomized clinical studies with RCC patients need to be conducted before cannabinoid receptor agonists could be introduced as potential therapeutic options for renal neoplasms.

## 7. Cholesterol in Renal Cancer

Cholesterol is a crucial component of cell membranes which plays an important role in the organization of lipid bilayers, being essential for membrane biogenesis and required for cell proliferation. Dietary intake is one source of cholesterol, but cholesterol is also synthesized by the liver and circulates throughout the body via low-density lipoprotein (LDL) and high-density lipoprotein (HDL) as carriers.

Several epidemiologic studies suggested a link between being overweight and obesity, and the risk of RCC [204,205,206]. Since the predominantly stored lipids in the cytoplasm of RCC are the cholesterol esters [207], it is of great importance to understand the regulation of cholesterol metabolism in RCC which could hold the key to novel treatment approaches [208]. Surprisingly, despite an increased intracellular accumulation of cholesterol in ccRCC cells, the genes encoding cholesterol biosynthetic enzymes are rather repressed suggesting that RCCs depend on extracellular cholesterol uptake for proliferation and survival [209]. The same authors observed that elevated levels of circulating high-density lipoprotein (HDL) cholesterol increase the risk of developing ccRCC and increased dietary cholesterol intake promotes tumor growth. Moreover, the preoperative serum total cholesterol level was shown to be a poor prognostic factor for patients with surgically treated RCC [210]. Given the association between obesity and increased RCC risk, the fact that obese patients frequently have elevated levels of cholesterol, and the intracellular cholesterol ester accumulation in RCC, further investigations to unravel the cholesterol metabolism alterations in RCC are needed in order to design new pharmacological targets.

In this regard, it should be noted that statins, as inhibitors of the 3-hydroxy-3-methylglutaryl-coenzyme A (HMG-CoA) reductase, are the most commonly prescribed drug class to reduce plasma cholesterol levels and, as a consequence, can lower the risk of cardiovascular events and mortality. Several observational studies reported that statins have a cancer-preventive effect in certain solid cancers, including RCC [211,212]. Over the years, many larger cohort studies, addressing the link between statin use and cancer risk, have been analyzed and subjected to meta-analyses, but still controversial conclusions remain. In this view, Luo et al. [213] performed a meta-analysis including 35 retrospective studies on the effect of statins in urologic cancers. They concluded that there is no benefit of statins in bladder cancers and RCC, except overall survival; the latter finding was probably derived from the protection from cardiovascular death. A similar conclusion was made by Wu et al. performing a meta-analysis from five studies including 5299 RCC [214].

## 8. Conclusions and Perspectives

**Figure 4:**
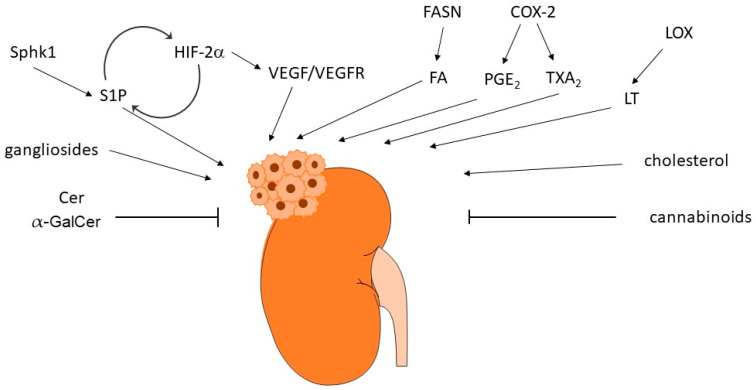
Summarizing scheme of bioactive lipids regulating renal cell carcinoma. Abbreviations: Cer, ceramide; COX-2, cyclooxygenase 2; FA, free fatty acid; FASN, fatty acid synthase; α-GalCer, galactosylceramide; HIF, hypoxia inducible factor; LOX, lipoxygenase; LT, leukotriene; PGE_2_, prostaglandin E_2_; S1P, sphingosine 1-phosphate; Sphk1, sphingosine kinase 1; TXA_2_, thromboxane A_2_; VEGF, vascular endothelium growth factor; VEGFR, VEGF receptor.

The reprograming of lipid metabolism is a typical characteristic feature of many tumors. Many of the so-far-described bioactive lipids can interfere with cancer-relevant molecular processes including cell proliferation and migration, apoptosis or survival, angiogenesis, and metastasis formation. Therefore, their involvement in RCC is very obvious (Figure 4) and this has been approached in multiple in vitro and in vivo studies over the last decades.

Another hallmark of RCC is the up-regulation of the HIF/VEGF signaling axis. It turns out that the targeting of the HIF/VEGF axis is very efficient in RCC treatment. Therefore, standard therapies to date are mainly focusing on blocking the VEGFR/tyrosine kinase receptor signaling in combination with inhibiting the immune cells. However, resistance development is a main problem, which stresses the need for better treatment options. Not surprisingly, many of the bioactive lipids are regulating HIF/VEGF signaling or the opposite, are themselves regulated by HIF/VEGF, which highlights the attractivity of the lipids as a new targeting strategy for RCC, and it will be exciting to see whether novel therapeutics can arise from these lipid cascades.

At least so far, none of the drugs tested in preclinical models have reached the clinical phase 3. While some of the drugs, such as sonepcizumab and NSAIDs, have failed in phase 2 clinical studies of RCC, others acting on more selective targets in the pathways, such as EP_4_ receptor antagonists or selective S1PR modulators, may turn out as the key. Additionally, lipids, such as the gangliosides or 15-LOX2 products, are involved in shaping the tumor microenvironment, and they might be important targets for immunotherapy (besides the standard immunotherapy for RCC). Finally, further research is needed in understanding the risk factors for RCC development such as the dietary fat intake.

Very recently, HIF-2α antagonists have been developed and tested positive in clinical trials for RCC. In 2021, the first inhibitor of this class, belzutifan, previously known as MK-6482 or PT2977, was FDA-approved for adult patients with VHL disease who require therapy for associated RCC. The most common adverse event was anemia occurring in 90% of the patients [215].

Another HIF-2α antagonist, PT2399, which is still in preclinical testing, also revealed positive effects in various RCC cell lines and in mouse models. It was also shown that PT2399 had greater activity than sunitinib, was active in sunitinib-progressing tumors, and was better tolerated. However, prolonged PT2399 treatment led to resistance [216].

Another important future step is the identification of reliable molecular biomarkers. Such biomarkers may either allow the early detection and diagnosis of RCC, or allow a prognosis on the risk of relapses and survival. Besides biomarkers obtained from tissue biopsies, urinary and blood biomarkers are becoming more and more attractive as they are more easily accessed than tissue biopsies. So far, reported biomarkers include circulating tumor cells (CTCs), cell-free DNA (cfDNA) in plasma/serum, several miRNAs, and metabolites in tissue, serum, and urine [217,218]. Very promising in these days is the approach of integrated transcriptomics, proteomics, and metabolomics [7,219,220], which will allow the identification of new biomarkers with high sensitivity and high precision.

## Data Availability

Not applicable.

## References

[1] Sung H., Ferlay J., Siegel R.L., Laversanne M., Soerjomataram I., Jemal A., Bray F. (2021). Global Cancer Statistics 2020: GLOBOCAN Estimates of Incidence and Mortality Worldwide for 36 Cancers in 185 Countries. CA Cancer J. Clin..

[2] Wu Q., Huang G., Wei W., Liu J. (2022). Molecular Imaging of Renal Cell Carcinoma in Precision Medicine. Mol. Pharm..

[3] Chow W.H., Dong L.M., Devesa S.S. (2010). Epidemiology and risk factors for kidney cancer. Nat. Rev. Urol..

[4] Moch H., Cubilla A.L., Humphrey P.A., Reuter V.E., Ulbright T.M. (2016). The 2016 WHO Classification of Tumours of the Urinary System and Male Genital Organs-Part A: Renal, Penile, and Testicular Tumours. Eur. Urol..

[5] Capitanio U., Bensalah K., Bex A., Boorjian S.A., Bray F., Coleman J., Gore J.L., Sun M., Wood C., Russo P. (2019). Epidemiology of Renal Cell Carcinoma. Eur. Urol..

[6] Drabkin H.A., Gemmill R.M. (2012). Cholesterol and the development of clear-cell renal carcinoma. Curr. Opin. Pharmacol..

[7] di Meo N.A., Lasorsa F., Rutigliano M., Loizzo D., Ferro M., Stella A., Bizzoca C., Vincenti L., Pandolfo S.D., Autorino R. (2022). Renal Cell Carcinoma as a Metabolic Disease: An Update on Main Pathways, Potential Biomarkers, and Therapeutic Targets. Int. J. Mol. Sci..

[8] Lucarelli G., Loizzo D., Franzin R., Battaglia S., Ferro M., Cantiello F., Castellano G., Bettocchi C., Ditonno P., Battaglia M. (2019). Metabolomic insights into pathophysiological mechanisms and biomarker discovery in clear cell renal cell carcinoma. Expert. Rev. Mol. Diagn..

[9] Latif F., Tory K., Gnarra J., Yao M., Duh F.M., Orcutt M.L., Stackhouse T., Kuzmin I., Modi W., Geil L. (1993). Identification of the von Hippel-Lindau disease tumor suppressor gene. Science.

[10] Gnarra J.R., Tory K., Weng Y., Schmidt L., Wei M.H., Li H., Latif F., Liu S., Chen F., Duh F.M. (1994). Mutations of the VHL tumour suppressor gene in renal carcinoma. Nat. Genet..

[11] Haase V.H. (2013). Regulation of erythropoiesis by hypoxia-inducible factors. Blood Rev..

[12] Wicks E.E., Semenza G.L. (2022). Hypoxia-inducible factors: Cancer progression and clinical translation. J. Clin. Investig..

[13] Patel S.A., Nilsson M.B., Le X., Cascone T., Jain R.K., Heymach J.V. (2023). Molecular Mechanisms and Future Implications of VEGF/VEGFR in Cancer Therapy. Clin. Cancer Res..

[14] Choueiri T.K., Kaelin W.G. (2020). Targeting the HIF2-VEGF axis in renal cell carcinoma. Nat. Med..

[15] Schirrmacher V. (2019). From chemotherapy to biological therapy: A review of novel concepts to reduce the side effects of systemic cancer treatment (Review). Int. J. Oncol..

[16] Wallace E.M., Rizzi J.P., Han G., Wehn P.M., Cao Z., Du X., Cheng T., Czerwinski R.M., Dixon D.D., Goggin B.S. (2016). A Small-Molecule Antagonist of HIF2alpha Is Efficacious in Preclinical Models of Renal Cell Carcinoma. Cancer Res..

[17] Semenza G.L. (1999). Regulation of mammalian O_2_ homeostasis by hypoxia-inducible factor 1. Annu. Rev. Cell Dev. Biol..

[18] Huwiler A., Kolter T., Pfeilschifter J., Sandhoff K. (2000). Physiology and pathophysiology of sphingolipid metabolism and signaling. Biochim. Biophys. Acta.

[19] Huwiler A., Pfeilschifter J. (2018). Sphingolipid signaling in renal fibrosis. Matrix Biol..

[20] Henry B., Moller C., Dimanche-Boitrel M.T., Gulbins E., Becker K.A. (2013). Targeting the ceramide system in cancer. Cancer Lett..

[21] Kim Y.J., Kim E.A., Sohn U.D., Yim C.B., Im C. (2010). Cytotoxic Activity and Structure Activity Relationship of Ceramide Analogues in Caki-2 and HL-60 Cells. Korean J. Physiol. Pharmacol..

[22] Stepanovska B., Huwiler A. (2020). Targeting the S1P receptor signaling pathways as a promising approach for treatment of autoimmune and inflammatory diseases. Pharmacol. Res..

[23] Takuwa Y., Du W., Qi X., Okamoto Y., Takuwa N., Yoshioka K. (2010). Roles of sphingosine-1-phosphate signaling in angiogenesis. World J. Biol. Chem..

[24] Hoefflin R., Harlander S., Abhari B.A., Peighambari A., Adlesic M., Seidel P., Zodel K., Haug S., Göcmen B., Li Y. (2021). Therapeutic Effects of Inhibition of Sphingosine-1-Phosphate Signaling in HIF-2α Inhibitor-Resistant Clear Cell Renal Cell Carcinoma. Cancers.

[25] Ader I., Brizuela L., Bouquerel P., Malavaud B., Cuvillier O. (2008). Sphingosine kinase 1: A new modulator of hypoxia inducible factor 1α during hypoxia in human cancer cells. Cancer Res..

[26] Bouquerel P., Gstalder C., Müller D., Laurent J., Brizuela L., Sabbadini R., Malavaud B., Pyronnet S., Martineau Y., Ader I. (2016). Essential role for SphK1/S1P signaling to regulate hypoxia-inducible factor 2α expression and activity in cancer. Oncogenesis.

[27] Anelli V., Gault C.R., Cheng A.B., Obeid L.M. (2008). Sphingosine kinase 1 is up-regulated during hypoxia in U87MG glioma cells: Role of hypoxia-inducible factors 1 and 2. J. Biol. Chem..

[28] Salama M.F., Carroll B., Adada M., Pulkoski-Gross M., Hannun Y.A., Obeid L.M. (2015). A novel role of sphingosine kinase-1 in the invasion and angiogenesis of VHL mutant clear cell renal cell carcinoma. FASEB J..

[29] Młynarczyk G., Mikłosz A., Suchański J., Reza S., Romanowicz L., Sobolewski K., Chabowski A., Baranowski M. (2022). Grade-dependent changes in sphingolipid metabolism in clear cell renal cell carcinoma. J. Cell. Biochem..

[30] Xu Y., Dong B., Wang J., Zhang J., Xue W., Huang Y. (2018). Sphingosine kinase 1 overexpression contributes to sunitinib resistance in clear cell renal cell carcinoma. Oncoimmunology.

[31] Cuvillier O., Ader I., Bouquerel P., Brizuela L., Gstalder C., Malavaud B. (2013). Hypoxia, therapeutic resistance, and sphingosine 1-phosphate. Adv. Cancer Res..

[32] Zhang L., Wang X., Bullock A.J., Callea M., Shah H., Song J., Moreno K., Visentin B., Deutschman D., Alsop D.C. (2015). Anti-S1P Antibody as a Novel Therapeutic Strategy for VEGFR TKI-Resistant Renal CancerS1P Inhibition as a New Treatment for RCC. Clin. Cancer Res..

[33] Huwiler A., Bourquin F., Kotelevets N., Pastukhov O., Capitani G., Grütter M.G., Zangemeister-Wittke U. (2011). A prokaryotic S1P lyase degrades extracellular S1P in vitro and in vivo: Implication for treating hyperproliferative disorders. PLoS ONE.

[34] O’Brien N., Jones S.T., Williams D.G., Cunningham H.B., Moreno K., Visentin B., Gentile A., Vekich J., Shestowsky W., Hiraiwa M. (2009). Production and characterization of monoclonal anti-sphingosine-1-phosphate antibodies 1. J. Lipid Res..

[35] Caballero S., Swaney J., Moreno K., Afzal A., Kielczewski J., Stoller G., Cavalli A., Garland W., Hansen G., Sabbadini R. (2009). Anti-sphingosine-1-phosphate monoclonal antibodies inhibit angiogenesis and sub-retinal fibrosis in a murine model of laser-induced choroidal neovascularization. Exp. Eye Res..

[36] Xie B., Shen J., Dong A., Rashid A., Stoller G., Campochiaro P.A. (2009). Blockade of sphingosine-1-phosphate reduces macrophage influx and retinal and choroidal neovascularization. J. Cell. Physiol..

[37] Pal S.K., Drabkin H.A., Reeves J.A., Hainsworth J.D., Hazel S.E., Paggiarino D.A., Wojciak J., Woodnutt G., Bhatt R.S. (2017). A phase 2 study of the sphingosine-1-phosphate antibody sonepcizumab in patients with metastatic renal cell carcinoma. Cancer.

[38] Huwiler A., Zangemeister-Wittke U. (2018). The sphingosine 1-phosphate receptor modulator fingolimod as a therapeutic agent: Recent findings and new perspectives. Pharmacol. Ther..

[39] Fischl A.S., Wang X., Falcon B.L., Almonte-Baldonado R., Bodenmiller D., Evans G., Stewart J., Wilson T., Hipskind P., Manro J. (2019). Inhibition of Sphingosine Phosphate Receptor 1 Signaling Enhances the Efficacy of VEGF Receptor InhibitionS1P1 Inhibition Improves VEGFR-Targeted Therapy. Mol. Cancer Ther..

[40] Ying Y., Ma X., Fang J., Chen S., Wang W., Li J., Xie H., Wu J., Xie B., Liu B. (2021). EGR2-mediated regulation of m6A reader IGF2BP proteins drive RCC tumorigenesis and metastasis via enhancing S1PR3 mRNA stabilization. Cell Death Dis..

[41] Yan Y., Bao G., Pei J., Cao Y., Zhang C., Zhao P., Zhang Y., Damirin A. (2022). NF-κB and EGFR participate in S1PR3-mediated human renal cell carcinomas progression. Biochim. Et Biophys. Acta (BBA)-Mol. Basis Dis..

[42] Glueck M., Koch A., Brunkhorst R., Ferreiros Bouzas N., Trautmann S., Schaefer L., Pfeilschifter W., Pfeilschifter J., Vutukuri R. (2022). The atypical sphingosine 1-phosphate variant, d16: 1 S1P, mediates CTGF induction via S1P2 activation in renal cell carcinoma. FEBS J..

[43] Hanada K. (2003). Serine palmitoyltransferase, a key enzyme of sphingolipid metabolism. Biochim. Et Biophys. Acta (BBA)-Mol. Cell Biol. Lipids.

[44] Zhu W.K., Xu W.H., Wang J., Huang Y.Q., Abudurexiti M., Qu Y.Y., Zhu Y.P., Zhang H.L., Ye D.W. (2020). Decreased SPTLC1 expression predicts worse outcomes in ccRCC patients. J. Cell. Biochem..

[45] Kong Z., Guo X., Zhao Z., Wu W., Luo L., Zhu Z., Yin S., Cai C., Wu W., Wang D. (2019). SPTLC1 inhibits cell growth via modulating Akt/FOXO1 pathway in renal cell carcinoma cells. Biochem. Biophys. Res. Commun..

[46] Wattenberg B.W. (2018). The long and the short of ceramides. J. Biol. Chem..

[47] Lee W.-K., Maaß M., Quach A., Poscic N., Prangley H., Pallott E.-C., Kim J.L., Pierce J.S., Ogretmen B., Futerman A.H. (2022). Dependence of ABCB1 transporter expression and function on distinct sphingolipids generated by ceramide synthases-2 and-6 in chemoresistant renal cancer. J. Biol. Chem..

[48] Schwaab T., Ernstoff M.S. (2011). Therapeutic vaccines in renal cell carcinoma. Therapy.

[49] Companioni O., Mir C., Garcia-Mayea Y., LLeonart M.E. (2021). Targeting Sphingolipids for Cancer Therapy. Front. Oncol..

[50] Lehmann N., Paret C., El Malki K., Russo A., Neu M.A., Wingerter A., Seidmann L., Foersch S., Ziegler N., Roth L. (2020). Tumor Lipids of Pediatric Papillary Renal Cell Carcinoma Stimulate Unconventional T Cells. Front. Immunol..

[51] Vyth-Dreese F.A., Sein J., van de Kasteele W., Dellemijn T.A., van den Bogaard C., Nooijen W.J., de Gast G.C., Haanen J.B., Bex A. (2010). Lack of anti-tumour reactivity despite enhanced numbers of circulating natural killer T cells in two patients with metastatic renal cell carcinoma. Clin. Exp. Immunol..

[52] Takahashi T., Suzuki T. (2012). Role of sulfatide in normal and pathological cells and tissues. J. Lipid Res..

[53] Sakakibara N. (1989). Glycolipid alterations in human kidney carcinoma. [Hokkaido Igaku Zasshi] Hokkaido J. Med. Sci..

[54] Sakakibara N., Gasa S., Kamio K., Makita A., Koyanagi T. (1989). Association of elevated sulfatides and sulfotransferase activities with human renal cell carcinoma. Cancer Res..

[55] Jirasko R., Idkowiak J., Wolrab D., Kvasnicka A., Friedecky D., Polanski K., Studentova H., Student V., Melichar B., Holcapek M. (2022). Altered Plasma, Urine, and Tissue Profiles of Sulfatides and Sphingomyelins in Patients with Renal Cell Carcinoma. Cancers.

[56] Porubsky S., Nientiedt M., Kriegmair M.C., Siemoneit J.-H.H., Sandhoff R., Jennemann R., Borgmann H., Gaiser T., Weis C.-A., Erben P. (2021). The prognostic value of galactosylceramide-sulfotransferase (Gal3ST1) in human renal cell carcinoma. Sci. Rep..

[57] Jirásko R., Holčapek M., Khalikova M., Vrána D., Študent V., Prouzová Z., Melichar B. (2017). MALDI orbitrap mass spectrometry profiling of dysregulated sulfoglycosphingolipids in renal cell carcinoma tissues. J. Am. Soc. Mass Spectrom..

[58] Liu Y.-Y., Patwardhan G.A., Xie P., Gu X., Giuliano A.E., Cabot M.C. (2011). Glucosylceramide synthase, a factor in modulating drug resistance, is overexpressed in metastatic breast carcinoma. Int. J. Oncol..

[59] Liu Y.-Y., Gupta V., Patwardhan G.A., Bhinge K., Zhao Y., Bao J., Mehendale H., Cabot M.C., Li Y.-T., Jazwinski S.M. (2010). Glucosylceramide synthase upregulates MDR1 expression in the regulation of cancer drug resistance through cSrc and β-catenin signaling. Mol. Cancer.

[60] Jones J., Otu H., Spentzos D., Kolia S., Inan M., Beecken W.D., Fellbaum C., Gu X., Joseph M., Pantuck A.J. (2005). Gene signatures of progression and metastasis in renal cell cancer. Clin. Cancer Res..

[61] Liu Y.-Y., Han T.Y., Yu J.Y., Bitterman A., Le A., Giuliano A.E., Cabot M.C. (2004). Oligonucleotides blocking glucosylceramide synthase expression selectively reverse drug resistance in cancer cells. J. Lipid Res..

[62] Chatterjee S., Alsaeedi N., Hou J., Bandaru V.V.R., Wu L., Halushka M.K., Pili R., Ndikuyeze G., Haughey N.J. (2013). Use of a glycolipid inhibitor to ameliorate renal cancer in a mouse model. PLoS ONE.

[63] Uzzo R.G., Rayman P., Kolenko V., Clark P.E., Cathcart M.K., Bloom T., Novick A.C., Bukowski R.M., Hamilton T., Finke J.H. (1999). Renal cell carcinoma–derived gangliosides suppress nuclear factor-κB activation in T cells. J. Clin. Investig..

[64] Finke J.H., Rayman P., George R., Tannenbaum C.S., Kolenko V., Uzzo R., Novick A.C., Bukowski R.M. (2001). Tumor-induced sensitivity to apoptosis in T cells from patients with renal cell carcinoma: Role of nuclear factor-κB suppression. Clin. Cancer Res..

[65] Kudo D., Rayman P., Horton C., Cathcart M.K., Bukowski R.M., Thornton M., Tannenbaum C., Finke J.H. (2003). Gangliosides expressed by the renal cell carcinoma cell line SK-RC-45 are involved in tumor-induced apoptosis of T cells. Cancer Res..

[66] Biswas S., Biswas K., Richmond A., Ko J., Ghosh S., Simmons M., Rayman P., Rini B., Gill I., Tannenbaum C.S. (2009). Elevated levels of select gangliosides in T cells from renal cell carcinoma patients is associated with T cell dysfunction. J. Immunol..

[67] Biswas K., Richmond A., Rayman P., Biswas S., Thornton M., Sa G., Das T., Zhang R., Chahlavi A., Tannenbaum C.S. (2006). GM2 expression in renal cell carcinoma: Potential role in tumor-induced T-cell dysfunction. Cancer Res..

[68] Hoon D.S., Okun E., Neuwirth H., Morton D.L., Irie R.F. (1993). Aberrant expression of gangliosides in human renal cell carcinomas. J. Urol..

[69] Ito A., Handa K., Withers D.A., Satoh M., Hakomori S.-i. (2001). Binding specificity of siglec7 to disialogangliosides of renal cell carcinoma: Possible role of disialogangliosides in tumor progression. FEBS Lett..

[70] Wu D.-Y., Adak A.K., Kuo Y.-T., Shen Y.-J., Li P.-J., Hwu J.R., Lin C.-C. (2020). A Modular Chemoenzymatic Synthesis of Disialosyl Globopentaosylceramide (DSGb5Cer) Glycan. J. Org. Chem..

[71] Kawasaki Y., Ito A., Kakoi N., Shimada S., Itoh J., Mitsuzuka K., Arai Y. (2015). Ganglioside, disialosyl globopentaosylceramide (DSGb5), enhances the migration of renal cell carcinoma cells. Tohoku J. Exp. Med..

[72] Itoh J., Ito A., Shimada S., Kawasaki Y., Kakoi N., Saito H., Mitsuzuka K., Watanabe M., Satoh M., Saito S. (2017). Clinicopathological significance of ganglioside DSGb5 expression in renal cell carcinoma. Glycoconj. J..

[73] Maruyama R., Saito S., Bilim V., Hara N., Itoi T., Yamana K., Nishiyama T., Arai Y., Takahashi K., Tomita Y. (2007). High incidence of GalNAc disialosyl lactotetraosylceramide in metastatic renal cell carcinoma. Anticancer. Res..

[74] Saito S., Orikasa S., Satoh M., Ohyama C., Ito A., Takahashi T. (1997). Expression of globo-series gangliosides in human renal cell carcinoma. Jpn. J. Cancer Res..

[75] Satoh M., Nejad F.M., Nakano O., Ito A., Kawamura S., Ohyama C., Saito S., Orikasa S. (1999). Four new human renal cell carcinoma cell lines expressing globo-series gangliosides. Tohoku J. Exp. Med..

[76] Tringali C., Lupo B., Silvestri I., Papini N., Anastasia L., Tettamanti G., Venerando B. (2012). The plasma membrane sialidase NEU3 regulates the malignancy of renal carcinoma cells by controlling β1 integrin internalization and recycling. J. Biol. Chem..

[77] Saito S., Nojiri H., Satoh M., Ito A., Ohyama C., Orikasa S. (2000). Inverse relationship of expression between GM3 and globo-series ganglioside in human renal cell carcinoma. Tohoku J. Exp. Med..

[78] Woutersen R.A., Appel M.J., van Garderen-Hoetmer A., Wijnands M.V. (1999). Dietary fat and carcinogenesis. Mutat. Res..

[79] Bojkova B., Winklewski P.J., Wszedybyl-Winklewska M. (2020). Dietary Fat and Cancer-Which Is Good, Which Is Bad, and the Body of Evidence. Int. J. Mol. Sci..

[80] Gerber M. (2012). Omega-3 fatty acids and cancers: A systematic update review of epidemiological studies. Br. J. Nutr..

[81] Liotti A., Cosimato V., Mirra P., Cali G., Conza D., Secondo A., Luongo G., Terracciano D., Formisano P., Beguinot F. (2018). Oleic acid promotes prostate cancer malignant phenotype via the G protein-coupled receptor FFA1/GPR40. J. Cell. Physiol..

[82] Brasky T.M., Darke A.K., Song X., Tangen C.M., Goodman P.J., Thompson I.M., Meyskens F.L., Goodman G.E., Minasian L.M., Parnes H.L. (2013). Plasma phospholipid fatty acids and prostate cancer risk in the SELECT trial. J. Natl. Cancer Inst..

[83] Liu Z., Xiao Y., Yuan Y., Zhang X., Qin C., Xie J., Hao Y., Xu T., Wang X. (2013). Effects of oleic acid on cell proliferation through an integrin-linked kinase signaling pathway in 786-O renal cell carcinoma cells. Oncol. Lett..

[84] Xiang F., Wu K., Liu Y., Shi L., Wang D., Li G., Tao K., Wang G. (2017). Omental adipocytes enhance the invasiveness of gastric cancer cells by oleic acid-induced activation of the PI3K-Akt signaling pathway. Int. J. Biochem. Cell Biol..

[85] Soto-Guzman A., Navarro-Tito N., Castro-Sanchez L., Martinez-Orozco R., Salazar E.P. (2010). Oleic acid promotes MMP-9 secretion and invasion in breast cancer cells. Clin. Exp. Metastasis.

[86] McCabe A.J., Wallace J.M., Gilmore W.S., McGlynn H., Strain S.J. (2005). Docosahexaenoic acid reduces in vitro invasion of renal cell carcinoma by elevated levels of tissue inhibitor of metalloproteinase-1. J. Nutr. Biochem..

[87] Bidoli E., Talamini R., Zucchetto A., Polesel J., Bosetti C., Negri E., Maruzzi D., Montella M., Franceschi S., La Vecchia C. (2008). Macronutrients, fatty acids, cholesterol and renal cell cancer risk. Int. J. Cancer.

[88] Brock K.E., Gridley G., Chiu B.C., Ershow A.G., Lynch C.F., Cantor K.P. (2009). Dietary fat and risk of renal cell carcinoma in the USA: A case-control study. Br. J. Nutr..

[89] Lee J.E., Spiegelman D., Hunter D.J., Albanes D., Bernstein L., van den Brandt P.A., Buring J.E., Cho E., English D.R., Freudenheim J.L. (2008). Fat, protein, and meat consumption and renal cell cancer risk: A pooled analysis of 13 prospective studies. J. Natl. Cancer Inst..

[90] Schwenk R.W., Holloway G.P., Luiken J.J., Bonen A., Glatz J.F. (2010). Fatty acid transport across the cell membrane: Regulation by fatty acid transporters. Prostaglandins Leukot Essent Fat. Acids.

[91] Schaffer J.E., Lodish H.F. (1994). Expression cloning and characterization of a novel adipocyte long chain fatty acid transport protein. Cell.

[92] Hara T., Kimura I., Inoue D., Ichimura A., Hirasawa A. (2013). Free fatty acid receptors and their role in regulation of energy metabolism. Rev. Physiol. Biochem. Pharmacol..

[93] Hopkins M.M., Meier K.E. (2017). Free Fatty Acid Receptors and Cancer: From Nutrition to Pharmacology. Handb. Exp. Pharmacol..

[94] Hopkins M.M., Meier K.E. (2017). Free fatty acid receptor (FFAR) agonists inhibit proliferation of human ovarian cancer cells. Prostaglandins Leukot Essent Fat. Acids.

[95] Hopkins M.M., Zhang Z., Liu Z., Meier K.E. (2016). Eicosopentaneoic Acid and Other Free Fatty Acid Receptor Agonists Inhibit Lysophosphatidic Acid- and Epidermal Growth Factor-Induced Proliferation of Human Breast Cancer Cells. J. Clin. Med..

[96] Wang J., Hong Y., Shao S., Zhang K., Hong W. (2018). FFAR1-and FFAR4-dependent activation of Hippo pathway mediates DHA-induced apoptosis of androgen-independent prostate cancer cells. Biochem. Biophys. Res. Commun..

[97] Takahashi K., Fukushima K., Onishi Y., Minami K., Otagaki S., Ishimoto K., Fukushima N., Honoki K., Tsujiuchi T. (2018). Involvement of FFA1 and FFA4 in the regulation of cellular functions during tumor progression in colon cancer cells. Exp. Cell Res..

[98] Monaco M.E. (2017). Fatty acid metabolism in breast cancer subtypes. Oncotarget.

[99] Tan S.K., Hougen H.Y., Merchan J.R., Gonzalgo M.L., Welford S.M. (2022). Fatty acid metabolism reprogramming in ccRCC: Mechanisms and potential targets. Nat. Rev. Urol..

[100] Alo P.L., Visca P., Marci A., Mangoni A., Botti C., Di Tondo U. (1996). Expression of fatty acid synthase (FAS) as a predictor of recurrence in stage I breast carcinoma patients. Cancer.

[101] Epstein J.I., Carmichael M., Partin A.W. (1995). OA-519 (fatty acid synthase) as an independent predictor of pathologic state in adenocarcinoma of the prostate. Urology.

[102] Rashid A., Pizer E.S., Moga M., Milgraum L.Z., Zahurak M., Pasternack G.R., Kuhajda F.P., Hamilton S.R. (1997). Elevated expression of fatty acid synthase and fatty acid synthetic activity in colorectal neoplasia. Am. J. Pathol..

[103] Nguyen P.L., Ma J., Chavarro J.E., Freedman M.L., Lis R., Fedele G., Fiore C., Qiu W., Fiorentino M., Finn S. (2010). Fatty acid synthase polymorphisms, tumor expression, body mass index, prostate cancer risk, and survival. J. Clin. Oncol..

[104] Horiguchi A., Asano T., Asano T., Ito K., Sumitomo M., Hayakawa M. (2008). Pharmacological inhibitor of fatty acid synthase suppresses growth and invasiveness of renal cancer cells. J. Urol..

[105] Xu W., Hu X., Anwaier A., Wang J., Liu W., Tian X., Zhu W., Ma C., Wan F., Shi G. (2020). Fatty Acid Synthase Correlates With Prognosis-Related Abdominal Adipose Distribution and Metabolic Disorders of Clear Cell Renal Cell Carcinoma. Front. Mol. Biosci..

[106] Yuan Y., Yang X., Li Y., Liu Q., Wu F., Qu H., Gao H., Ge J., Xu Y., Wang H. (2020). Expression and prognostic significance of fatty acid synthase in clear cell renal cell carcinoma. Pathol. Res. Pract..

[107] Che L., Paliogiannis P., Cigliano A., Pilo M.G., Chen X., Calvisi D.F. (2019). Pathogenetic, Prognostic, and Therapeutic Role of Fatty Acid Synthase in Human Hepatocellular Carcinoma. Front. Oncol..

[108] Lu T., Sun L., Wang Z., Zhang Y., He Z., Xu C. (2019). Fatty acid synthase enhances colorectal cancer cell proliferation and metastasis via regulating AMPK/mTOR pathway. Onco Targets Ther..

[109] Chang L., Fang S., Chen Y., Yang Z., Yuan Y., Zhang J., Ye L., Gu W. (2019). Inhibition of FASN suppresses the malignant biological behavior of non-small cell lung cancer cells via deregulating glucose metabolism and AKT/ERK pathway. Lipids Health Dis..

[110] Wettersten H.I., Hakimi A.A., Morin D., Bianchi C., Johnstone M.E., Donohoe D.R., Trott J.F., Aboud O.A., Stirdivant S., Neri B. (2015). Grade-Dependent Metabolic Reprogramming in Kidney Cancer Revealed by Combined Proteomics and Metabolomics Analysis. Cancer Res..

[111] Albiges L., Hakimi A.A., Xie W., McKay R.R., Simantov R., Lin X., Lee J.L., Rini B.I., Srinivas S., Bjarnason G.A. (2016). Body Mass Index and Metastatic Renal Cell Carcinoma: Clinical and Biological Correlations. J. Clin. Oncol..

[112] Qiu Z., Huang C., Sun J., Qiu W., Zhang J., Li H., Jiang T., Huang K., Cao J. (2007). RNA interference-mediated signal transducers and activators of transcription 3 gene silencing inhibits invasion and metastasis of human pancreatic cancer cells. Cancer Sci..

[113] Slade R.F., Hunt D.A., Pochet M.M., Venema V.J., Hennigar R.A. (2003). Characterization and inhibition of fatty acid synthase in pediatric tumor cell lines. Anticancer. Res..

[114] Wang X., Du G., Wu Y., Zhang Y., Guo F., Liu W., Wu R. (2020). Association between different levels of lipid metabolismrelated enzymes and fatty acid synthase in Wilms’ tumor. Int. J. Oncol..

[115] Camassei F.D., Jenkner A., Rava L., Bosman C., Francalanci P., Donfrancesco A., Alo P.L., Boldrini R. (2003). Expression of the lipogenic enzyme fatty acid synthase (FAS) as a predictor of poor outcome in nephroblastoma: An interinstitutional study. Med. Pediatr. Oncol..

[116] Quan J., Bode A.M., Luo X. (2021). ACSL family: The regulatory mechanisms and therapeutic implications in cancer. Eur. J. Pharmacol..

[117] Cancer Genome Atlas Research N. (2013). Comprehensive molecular characterization of clear cell renal cell carcinoma. Nature.

[118] Zhang C.Y., Hu H.L., Huang R.Z., Huang G.M., Xi X.Q. (2022). ACSL3 is a potential prognostic biomarker for immune infiltration in clear cell renal cell carcinoma. Front. Surg..

[119] Klasson T.D., LaGory E.L., Zhao H., Huynh S.K., Papandreou I., Moon E.J., Giaccia A.J. (2022). ACSL3 regulates lipid droplet biogenesis and ferroptosis sensitivity in clear cell renal cell carcinoma. Cancer Metab..

[120] Zhang Y., Wang H., Zhang J., Lv J., Huang Y. (2013). Positive feedback loop and synergistic effects between hypoxia-inducible factor-2alpha and stearoyl-CoA desaturase-1 promote tumorigenesis in clear cell renal cell carcinoma. Cancer Sci..

[121] von Roemeling C.A., Marlow L.A., Wei J.J., Cooper S.J., Caulfield T.R., Wu K., Tan W.W., Tun H.W., Copland J.A. (2013). Stearoyl-CoA desaturase 1 is a novel molecular therapeutic target for clear cell renal cell carcinoma. Clin. Cancer Res..

[122] Tracz-Gaszewska Z., Dobrzyn P. (2019). Stearoyl-CoA Desaturase 1 as a Therapeutic Target for the Treatment of Cancer. Cancers.

[123] Budhu A., Roessler S., Zhao X., Yu Z., Forgues M., Ji J., Karoly E., Qin L.X., Ye Q.H., Jia H.L. (2013). Integrated metabolite and gene expression profiles identify lipid biomarkers associated with progression of hepatocellular carcinoma and patient outcomes. Gastroenterology.

[124] Chavarro J.E., Kenfield S.A., Stampfer M.J., Loda M., Campos H., Sesso H.D., Ma J. (2013). Blood levels of saturated and monounsaturated fatty acids as markers of de novo lipogenesis and risk of prostate cancer. Am. J. Epidemiol..

[125] Raeisi M., Hassanbeigi L., Khalili F., Kharrati-Shishavan H., Yousefi M., Mehdizadeh A. (2022). Stearoyl-CoA desaturase 1 as a therapeutic target for cancer: A focus on hepatocellular carcinoma. Mol. Biol. Rep..

[126] Calder P.C. (2020). Eicosanoids. Essays Biochem..

[127] Gomes R.N., Felipe da Costa S., Colquhoun A. (2018). Eicosanoids and cancer. Clinics.

[128] Wang D., DuBois R.N. (2010). Eicosanoids and cancer. Nat. Rev. Cancer.

[129] Wang Q., Morris R.J., Bode A.M., Zhang T. (2022). Prostaglandin pathways: Opportunities for cancer prevention and therapy. Cancer Res..

[130] De Keijzer S., Meddens M.B., Torensma R., Cambi A. (2013). The multiple faces of prostaglandin E2 G-protein coupled receptor signaling during the dendritic cell life cycle. Int. J. Mol. Sci..

[131] Harris R.C. (2006). COX-2 and the kidney. J. Cardiovasc. Pharmacol..

[132] Tuna B., Yorukoglu K., Gurel D., Mungan U., Kirkali Z. (2004). Significance of COX-2 expression in human renal cell carcinoma. Urology.

[133] Osman W.M., Youssef N.S. (2015). Combined use of COX-1 and VEGF immunohistochemistry refines the histopathologic prognosis of renal cell carcinoma. Int. J. Clin. Exp. Pathol..

[134] Miyata Y., Koga S., Kanda S., Nishikido M., Hayashi T., Kanetake H. (2003). Expression of cyclooxygenase-2 in renal cell carcinoma: Correlation with tumor cell proliferation, apoptosis, angiogenesis, expression of matrix metalloproteinase-2, and survival. Clin. Cancer Res..

[135] Cho D.S., Joo H.J., Oh D.K., Kang J.H., Kim Y.S., Lee K.B., Kim S.J. (2005). Cyclooxygenase-2 and p53 expression as prognostic indicators in conventional renal cell carcinoma. Yonsei Med. J..

[136] Kankuri-Tammilehto M.K., Söderström K.-O., Pelliniemi T.-T., Vahlberg T., Pyrhönen S.O., Salminen E.K. (2010). Prognostic evaluation of COX-2 expression in renal cell carcinoma. Anticancer. Res..

[137] Hashemi Goradel N., Najafi M., Salehi E., Farhood B., Mortezaee K. (2019). Cyclooxygenase-2 in cancer: A review. J. Cell. Physiol..

[138] Park G., Song N.-Y., Kim D.-H., Lee S.-J., Chun K.-S. (2021). Thymoquinone suppresses migration of human renal carcinoma caki-1 cells through inhibition of the PGE2-mediated activation of the EP2 receptor pathway. Biomol. Ther..

[139] Liu B., Qu L., Yan S. (2015). Cyclooxygenase-2 promotes tumor growth and suppresses tumor immunity. Cancer Cell Int..

[140] Li J., Feng G., Liu J., Rong R., Luo F., Guo L., Zhu T., Wang G., Chu Y. (2010). Renal cell carcinoma may evade the immune system by converting CD4+ Foxp3-T cells into CD4+ CD25+ Foxp3+ regulatory T cells: Role of tumor COX-2-derived PGE2. Mol. Med. Rep..

[141] Li J.F., Chu Y.W., Wang G.M., Zhu T.Y., Rong R.M., Hou J., Xu M. (2009). The prognostic value of peritumoral regulatory T cells and its correlation with intratumoral cyclooxygenase-2 expression in clear cell renal cell carcinoma. BJU Int..

[142] Smyth G.P., Stapleton P.P., Barden C.B., Mestre J.R., Freeman T.A., Duff M.D., Maddali S., Yan Z., Daly J.M. (2003). Renal cell carcinoma induces prostaglandin E2 and T-helper type 2 cytokine production in peripheral blood mononuclear cells. Ann. Surg. Oncol..

[143] Menetrier-Caux C., Bain C., Favrot M., Duc A., Blay J. (1999). Renal cell carcinoma induces interleukin 10 and prostaglandin E2 production by monocytes. Br. J. Cancer.

[144] Hamieh L., Moreira R.B., Lin X., Simantov R., Choueiri T.K., McKay R.R. (2018). Impact of Aspirin and Non-Aspirin Nonsteroidal Anti-Inflammatory Drugs on Outcomes in Patients with Metastatic Renal Cell Carcinoma. Kidney Cancer.

[145] Ohba K., Miyata Y., Sakai H. (2013). Expression and function of E prostanoid receptors in urological cancer. Hinyokika Kiyo. Acta Urol. Jpn..

[146] Sato N., Mizutani Y., Li Y.N., Fujiwara J., Ishida H., Toiyama D., Abe K., Hayashi I., Nakanishi H., Kawauchi A. (2010). Enhancement of the sensitivity of renal cell carcinoma cells to fas-mediated cytotoxicity and apoptosis by the selective cyclooxygenase-2 inhibitor JTE-522. Urol. Int..

[147] Yoshimura R., Matsuyama M., Kawahito Y., Takemoto Y., Tsuchida K., Kuratsukuri K., Segawa Y., Shinnka T., Sano H., Nakatani T. (2004). The effects of cyclooxygenase-2 inhibitors on urological cancer cells. Int. J. Mol. Med..

[148] Wang X., Zhang L., O’neill A., Bahamon B., Alsop D.C., Mier J.W., Goldberg S.N., Signoretti S., Atkins M., Wood C. (2013). Cox-2 inhibition enhances the activity of sunitinib in human renal cell carcinoma xenografts. Br. J. Cancer.

[149] Shinohara N., Kumagai A., Kanagawa K., Maruyama S., Abe T., Sazawa A., Nonomura K. (2009). Multicenter phase II trial of combination therapy with meloxicam, a COX-2 inhibitor, and natural interferon-α for metastatic renal cell carcinoma. Jpn. J. Clin. Oncol..

[150] Rini B.I., Weinberg V., Dunlap S., Elchinoff A., Yu N., Bok R., Simko J., Small E.J. (2006). Maximal COX-2 immunostaining and clinical response to celecoxib and interferon alpha therapy in metastatic renal cell carcinoma. Cancer Interdiscip. Int. J. Am. Cancer Soc..

[151] Schwandt A., Garcia J.A., Elson P., Wyckhouse J., Finke J.H., Ireland J., Triozzi P., Zhou M., Dreicer R., Rini B.I. (2011). Clinical and immunomodulatory effects of celecoxib plus interferon-alpha in metastatic renal cell carcinoma patients with COX-2 tumor immunostaining. J. Clin. Immunol..

[152] Tatokoro M., Fujii Y., Kawakami S., Saito K., Koga F., Matsuoka Y., Iimura Y., Masuda H., Kihara K. (2011). Phase-II trial of combination treatment of interferon-α, cimetidine, cyclooxygenase-2 inhibitor and renin-angiotensin-system inhibitor (I-CCA therapy) for advanced renal cell carcinoma. Cancer Sci..

[153] Luo L., Liang Y., Ding X., Ma X., Zhang G., Sun L. (2019). Significance of cyclooxygenase-2, prostaglandin E2 and CD133 levels in sunitinib-resistant renal cell carcinoma. Oncol. Lett..

[154] Ohba K., Miyata Y., Watanabe S.-I., Hayashi T., Kanetake H., Kanda S., Sakai H. (2011). Clinical significance and predictive value of prostaglandin E2 receptors (EPR) 1–4 in patients with renal cell carcinoma. Anticancer. Res..

[155] Wu J., Zhang Y., Frilot N., Kim J.I., Kim W.-J., Daaka Y. (2011). Prostaglandin E2 regulates renal cell carcinoma invasion through the EP4 receptor-Rap GTPase signal transduction pathway. J. Biol. Chem..

[156] Zhang Y., Purayil H.T., Black J.B., Fetto F., Lynch L.D., Masannat J.N., Daaka Y. (2017). Prostaglandin E2 receptor 4 mediates renal cell carcinoma intravasation and metastasis. Cancer Lett..

[157] Li Z., Zhang Y., Kim W., Daaka Y. (2013). PGE2 promotes renal carcinoma cell invasion through activated RalA. Oncogene.

[158] Chen Q., Shinohara N., Abe T., Harabayashi T., Nonomura K. (2004). Impact of cyclooxygenase-2 gene expression on tumor invasiveness in a human renal cell carcinoma cell line. J. Urol..

[159] Hong D.S., Parikh A., Shapiro G.I., Varga A., Naing A., Meric-Bernstam F., Ataman Ö., Reyderman L., Binder T.A., Ren M. (2020). First-in-human phase I study of immunomodulatory E7046, an antagonist of PGE2-receptor E-type 4 (EP4), in patients with advanced cancers. J. Immunother. Cancer.

[160] Take Y., Koizumi S., Nagahisa A. (2020). Prostaglandin E receptor 4 antagonist in cancer immunotherapy: Mechanisms of action. Front. Immunol..

[161] Remuzzi G., FitzGerald G.A., Patrono C. (1992). Thromboxane synthesis and action within the kidney. Kidney Int..

[162] Ashton A.W., Zhang Y., Cazzolli R., Honn K.V. (2022). The Role and Regulation of Thromboxane A(2) Signaling in Cancer-Trojan Horses and Misdirection. Molecules.

[163] Moussa O., Ashton A.W., Fraig M., Garrett-Mayer E., Ghoneim M.A., Halushka P.V., Watson D.K. (2008). Novel role of thromboxane receptors β isoform in bladder cancer pathogenesis. Cancer Res..

[164] Faronato M., Muzzonigro G., Milanese G., Menna C., Bonfigli A., Catalano A., Procopio A. (2007). Increased expression of 5-lipoxygenase is common in clear cell renal cell carcinoma. Histol. Histopathol..

[165] Matsuyama M., Yoshimura R., Mitsuhashi M., Tsuchida K., Takemoto Y., Kawahito Y., Sano H., Nakatani T. (2005). 5-Lipoxygenase inhibitors attenuate growth of human renal cell carcinoma and induce apoptosis through arachidonic acid pathway. Oncol. Rep..

[166] Matsuyama M., Yoshimura R. (2008). Relationship between arachidonic acid pathway and human renal cell carcinoma. OncoTargets Ther..

[167] Selka A., Doiron J.A., Lyons P., Dastous S., Chiasson A., Cormier M., Turcotte S., Surette M.E., Touaibia M. (2019). Discovery of a novel 2, 5-dihydroxycinnamic acid-based 5-lipoxygenase inhibitor that induces apoptosis and may impair autophagic flux in RCC4 renal cancer cells. Eur. J. Med. Chem..

[168] Thalanayar Muthukrishnan P., Nouraie M., Parikh A., Holguin F. (2020). Zileuton use and phenotypic features in asthma. Pulm. Pharmacol. Ther..

[169] Zhang X., Wu H., Yan X., Ma J., Chen Z. (2022). LTB4R Promotes the Occurrence and Progression of Clear Cell Renal Cell Carcinoma (ccRCC) by Regulating the AKT/mTOR Signaling Pathway. Cells.

[170] Wu H.-H., Yan X., Chen Z., Du G.-W., Bai X.-J., Tuoheti K., Liu T.-Z. (2021). GNRH1 and LTB4R might be novel immune-related prognostic biomarkers in clear cell renal cell carcinoma (ccRCC). Cancer Cell Int..

[171] Yuan X., He Y., Luo C., Wang W. (2022). Leukotriene B4 receptor 2 correlates with prognosis and immune infiltration in clear cell renal cell carcinoma. Investig. New Drugs.

[172] Funao K., Matsuyama M., Naganuma T., Kawahito Y., Sano H., Nakatani T., Yoshimura R. (2008). The cysteinylLT1 receptor in human renal cell carcinoma. Mol. Med. Rep..

[173] Matsuyama M., Yoshimura R. (2010). Cysteinyl-leukotriene1 receptor is a potent target for the prevention and treatment of human urological cancer. Mol. Med. Rep..

[174] Tsai M.-J., Wu P.-H., Sheu C.-C., Hsu Y.-L., Chang W.-A., Hung J.-Y., Yang C.-J., Yang Y.-H., Kuo P.-L., Huang M.-S. (2016). Cysteinyl leukotriene receptor antagonists decrease cancer risk in asthma patients. Sci. Rep..

[175] Wolf C., Smith S., van Wijk S.J. (2022). Zafirlukast Induces VHL-and HIF-2α-Dependent Oxidative Cell Death in 786-O Clear Cell Renal Carcinoma Cells. Int. J. Mol. Sci..

[176] Yoshimura R., Inoue K., Kawahito Y., Mitsuhashi M., Tsuchida K., Matsuyama M., Sano H., Nakatani T. (2004). Expression of 12-lipoxygenase in human renal cell carcinoma and growth prevention by its inhibitor. Int. J. Mol. Med..

[177] Daurkin I., Eruslanov E., Stoffs T., Perrin G.Q., Algood C., Gilbert S.M., Rosser C.J., Su L.-M., Vieweg J., Kusmartsev S. (2011). Tumor-Associated Macrophages Mediate Immunosuppression in the Renal Cancer Microenvironment by Activating the 15-Lipoxygenase-2 PathwayTumor-Associated Macrophages in Human Kidney Cancer. Cancer Res..

[178] Kusmartsev S. (2012). Enhanced 15-lipoxygenase activity and elevated eicosanoid production in kidney tumor microenvironment contribute to the inflammation and immune suppression. Oncoimmunology.

[179] Gohara A., Eltaki N., Sabry D., Murtagh D., Jankun J., Selman S.H., Skrzypczak-Jankun E. (2012). Human 5-, 12-and 15-lipoxygenase-1 coexist in kidney but show opposite trends and their balance changes in cancer. Oncol. Rep..

[180] Alexanian A., Sorokin A. (2013). Targeting 20-HETE producing enzymes in cancer–rationale, pharmacology, and clinical potential. OncoTargets Ther..

[181] Alexanian A., Rufanova V.A., Miller B., Flasch A., Roman R.J., Sorokin A. (2009). Down-regulation of 20-HETE synthesis and signaling inhibits renal adenocarcinoma cell proliferation and tumor growth. Anticancer. Res..

[182] Liu J., Wang L., Harvey-White J., Osei-Hyiaman D., Razdan R., Gong Q., Chan A.C., Zhou Z., Huang B.X., Kim H.Y. (2006). A biosynthetic pathway for anandamide. Proc. Natl. Acad. Sci. USA.

[183] Simon G.M., Cravatt B.F. (2008). Anandamide biosynthesis catalyzed by the phosphodiesterase GDE1 and detection of glycerophospho-N-acyl ethanolamine precursors in mouse brain. J. Biol. Chem..

[184] Maccarrone M. (2016). Need for Methods to Investigate Endocannabinoid Signaling. Methods Mol. Biol..

[185] Pisanti S., Bifulco M. (2009). Endocannabinoid system modulation in cancer biology and therapy. Pharmacol. Res..

[186] Sanchez C., Galve-Roperh I., Canova C., Brachet P., Guzman M. (1998). Delta9-tetrahydrocannabinol induces apoptosis in C6 glioma cells. FEBS Lett..

[187] Elbaz M., Ahirwar D., Ravi J., Nasser M.W., Ganju R.K. (2017). Novel role of cannabinoid receptor 2 in inhibiting EGF/EGFR and IGF-I/IGF-IR pathways in breast cancer. Oncotarget.

[188] Ruiz L., Miguel A., Diaz-Laviada I. (1999). Delta9-tetrahydrocannabinol induces apoptosis in human prostate PC-3 cells via a receptor-independent mechanism. FEBS Lett..

[189] Maccarrone M., Lorenzon T., Bari M., Melino G., Finazzi-Agro A. (2000). Anandamide induces apoptosis in human cells via vanilloid receptors. Evidence for a protective role of cannabinoid receptors. J. Biol. Chem..

[190] Petrosino S., Di Marzo V. (2010). FAAH and MAGL inhibitors: Therapeutic opportunities from regulating endocannabinoid levels. Curr. Opin. Investig. Drugs.

[191] Hu W.R., Lian Y.F., Peng L.X., Lei J.J., Deng C.C., Xu M., Feng Q.S., Chen L.Z., Bei J.X., Zeng Y.X. (2014). Monoacylglycerol lipase promotes metastases in nasopharyngeal carcinoma. Int. J. Clin. Exp. Pathol..

[192] Gong X., Zheng X., Huang Y., Song W., Chen G., Chen T. (2022). Monoacylglycerol Lipase (MAGL) Inhibition Impedes the Osteosarcoma Progression by Regulating Epithelial Mesenchymal Transition. Tohoku J. Exp. Med..

[193] Endsley M.P., Thill R., Choudhry I., Williams C.L., Kajdacsy-Balla A., Campbell W.B., Nithipatikom K. (2008). Expression and function of fatty acid amide hydrolase in prostate cancer. Int. J. Cancer.

[194] Winkler K., Ramer R., Dithmer S., Ivanov I., Merkord J., Hinz B. (2016). Fatty acid amide hydrolase inhibitors confer anti-invasive and antimetastatic effects on lung cancer cells. Oncotarget.

[195] Izzo A.A., Aviello G., Petrosino S., Orlando P., Marsicano G., Lutz B., Borrelli F., Capasso R., Nigam S., Capasso F. (2008). Increased endocannabinoid levels reduce the development of precancerous lesions in the mouse colon. J. Mol. Med..

[196] Larrinaga G., Sanz B., Perez I., Blanco L., Candenas M.L., Pinto F.M., Gil J., Lopez J.I. (2010). Cannabinoid CB(1) receptor is downregulated in clear cell renal cell carcinoma. J. Histochem. Cytochem..

[197] Wang J., Xu Y., Zhu L., Zou Y., Kong W., Dong B., Huang J., Chen Y., Xue W., Huang Y. (2018). Cannabinoid receptor 2 as a novel target for promotion of renal cell carcinoma prognosis and progression. J. Cancer Res. Clin. Oncol..

[198] Khan M.I., Sobocinska A.A., Brodaczewska K.K., Zielniok K., Gajewska M., Kieda C., Czarnecka A.M., Szczylik C. (2018). Involvement of the CB2 cannabinoid receptor in cell growth inhibition and G0/G1 cell cycle arrest via the cannabinoid agonist WIN 55,212-2 in renal cell carcinoma. BMC Cancer.

[199] Tang M., Cao X., Zhang K., Li Y., Zheng Q.Y., Li G.Q., He Q.H., Li S.J., Xu G.L., Zhang K.Q. (2018). Celastrol alleviates renal fibrosis by upregulating cannabinoid receptor 2 expression. Cell Death Dis..

[200] Jiang X., Chen S., Zhang Q., Yi C., He J., Ye X., Liu M., Lu W. (2020). Celastrol is a novel selective agonist of cannabinoid receptor 2 with anti-inflammatory and anti-fibrotic activity in a mouse model of systemic sclerosis. Phytomedicine.

[201] Croxford J.L., Yamamura T. (2005). Cannabinoids and the immune system: Potential for the treatment of inflammatory diseases?. J. Neuroimmunol..

[202] Katz-Talmor D., Katz I., Porat-Katz B.S., Shoenfeld Y. (2018). Cannabinoids for the treatment of rheumatic diseases-where do we stand?. Nat. Rev. Rheumatol..

[203] Taha T., Meiri D., Talhamy S., Wollner M., Peer A., Bar-Sela G. (2019). Cannabis Impacts Tumor Response Rate to Nivolumab in Patients with Advanced Malignancies. Oncologist.

[204] Samanic C., Chow W.H., Gridley G., Jarvholm B., Fraumeni J.F. (2006). Relation of body mass index to cancer risk in 362,552 Swedish men. Cancer Causes Control.

[205] Adams K.F., Leitzmann M.F., Albanes D., Kipnis V., Moore S.C., Schatzkin A., Chow W.H. (2008). Body size and renal cell cancer incidence in a large US cohort study. Am. J. Epidemiol..

[206] Reeves G.K., Pirie K., Beral V., Green J., Spencer E., Bull D., Million Women Study C. (2007). Cancer incidence and mortality in relation to body mass index in the Million Women Study: Cohort study. BMJ.

[207] Gebhard R.L., Clayman R.V., Prigge W.F., Figenshau R., Staley N.A., Reesey C., Bear A. (1987). Abnormal cholesterol metabolism in renal clear cell carcinoma. J. Lipid Res..

[208] Heravi G., Yazdanpanah O., Podgorski I., Matherly L.H., Liu W. (2022). Lipid metabolism reprogramming in renal cell carcinoma. Cancer Metastasis Rev..

[209] Riscal R., Bull C.J., Mesaros C., Finan J.M., Carens M., Ho E.S., Xu J.P., Godfrey J., Brennan P., Johansson M. (2021). Cholesterol Auxotrophy as a Targetable Vulnerability in Clear Cell Renal Cell Carcinoma. Cancer Discov..

[210] Li B., Huang D., Zheng H., Cai Q., Guo Z., Wang S. (2020). Preoperative serum total cholesterol is a predictor of prognosis in patients with renal cell carcinoma: A meta- analysis of observational studies. Int. Braz. J. Urol..

[211] Santoni M., Monteiro F.S.M., Massari F., Abahssain H., Aurilio G., Molina-Cerrillo J., Myint Z.W., Zabalza I.O., Battelli N., Grande E. (2022). Statins and renal cell carcinoma: Antitumor activity and influence on cancer risk and survival. Crit. Rev. Oncol. Hematol..

[212] Nielsen S.F., Nordestgaard B.G., Bojesen S.E. (2012). Statin use and reduced cancer-related mortality. N. Engl. J. Med..

[213] Luo Y., She D.L., Xiong H., Fu S.J., Yang L. (2015). The Prognostic Effect of Statin Use on Urologic Cancers: An Updated Meta-Analysis of 35 Observational Studies. Medicine.

[214] Wu P., Xiang T., Wang J., Lv R., Zhuang Y., Wu G. (2020). Statin use and the overall survival of renal cell carcinoma: A meta-analysis. Clin. Investig. Med..

[215] Jonasch E., Donskov F., Iliopoulos O., Rathmell W.K., Narayan V.K., Maughan B.L., Oudard S., Else T., Maranchie J.K., Welsh S.J. (2021). Belzutifan for Renal Cell Carcinoma in von Hippel-Lindau Disease. N. Engl. J. Med..

[216] Chen W., Hill H., Christie A., Kim M.S., Holloman E., Pavia-Jimenez A., Homayoun F., Ma Y., Patel N., Yell P. (2016). Targeting renal cell carcinoma with a HIF-2 antagonist. Nature.

[217] Kapoor J., Claps F., Mir M.C., Ischia J. (2021). Promising Biomarkers in Renal Cell Carcinoma. Soc. Int. Urol. J..

[218] Claps F., Mir M.C. (2021). Novel Expanding Renal Cell Carcinoma Biomarkers. Soc. Int. Urol. J..

[219] Lucarelli G., Rutigliano M., Sallustio F., Ribatti D., Giglio A., Signorile M.L., Grossi V., Sanese P., Napoli A., Maiorano E. (2018). Integrated multi-omics characterization reveals a distinctive metabolic signature and the role of NDUFA4L2 in promoting angiogenesis, chemoresistance, and mitochondrial dysfunction in clear cell renal cell carcinoma. Aging (Albany NY).

[220] Ragone R., Sallustio F., Piccinonna S., Rutigliano M., Vanessa G., Palazzo S., Lucarelli G., Ditonno P., Battaglia M., Fanizzi F.P. (2016). Renal Cell Carcinoma: A Study through NMR-Based Metabolomics Combined with Transcriptomics. Diseases.

